# XPNPEP2 regulates angiogenesis via modulation of mitochondrial function through SLC25A6

**DOI:** 10.3389/fcell.2025.1698651

**Published:** 2026-01-07

**Authors:** Chenxi Yang, Yijun Lu, Yu Xia, Bingying Wang, Jie Xu, Yuchen Zhang, Jiaxuan Yan, Min Liu, Ting Chen, Xiaoxu Zhao, Xiaohui Cang, Jianhua Mao, Pingping Jiang

**Affiliations:** 1 Department of Nephrology, National Clinical Research Center for Child Health, Children’s Hospital, Zhejiang University School of Medicine, Hangzhou, China; 2 Assisted Reproduction Center, Northwest Women’s and Children’s Hospital, Xi’an, China; 3 Zhejiang Provincial Key Laboratory of Synthetic Biotechnology for Microbial Medicine, Zhejiang University School of Medicine, Hangzhou, China; 4 Zhejiang University School of Medicine, Hangzhou, China; 5 Medical Science and Technology Innovation Center, Shandong First Medical University & Shandong Academy of Medical Sciences, Jinan, China

**Keywords:** interaction of SLC25A6, mitochondria, mitochondria-associated membranes, neoangiogenesis, Siah E3 ubiquitin protein ligase 1, X-prolyl aminopeptidase 2

## Abstract

X-prolyl aminopeptidase 2 (XPNPEP2), which is abundantly expressed in vascular endothelial cells (ECs), has been reported to be associated with cardiovascular disease and angiogenesis. However, its function in ECs and its involvement in the pathogenesis of angiogenesis remain unclear. In this study, we revealed that XPNPEP2 is essential for EC function and angiogenesis via modulation of mitochondrial function. *In vivo*, XPNPEP2 deletion led to pathological changes in the pulmonary artery wall and renal tissue, decreased venous blood vessel density in the proximal region of superficial retinal vessels, and significantly slowed wound healing and tumor growth in mice. *In vitro*, XPNPEP2 deficiency impaired EC proliferation, migration, and tubulogenesis, which was accompanied by diminished mitochondria-associated membranes and dysfunctional mitochondria, including insufficient ATP, excessive mitochondrial reactive oxygen species (mROS), and disrupted respiration chain function. XPNPEP2 was found to interact with SLC25A6. The overexpression of XPNPEP2 restored impaired EC angiogenesis and the reduction in SLC25A6 caused by XPNPEP2 ablation. Moreover, inhibition of XPNPEP2 downregulated SLC25A6 via Siah E3 ubiquitin protein ligase 1 (SIAH1)-mediated degradation. Additionally, attenuated EC angiogenesis was achieved solely by silencing SLC25A6. Our findings highlight that XPNPEP2 regulates angiogenesis via modulation of mitochondrial function, which may represent a new strategy for the treatment of angiogenesis-related diseases.

## 1 Introduction

Angiogenesis, the capacity of the vasculature to form new blood vessels, is crucial for growth and development, tissue and organ remodeling, and even numerous pathological conditions (Eelen et al., 2015). Under proangiogenic signals, such as vascular endothelial growth factor (VEGF) (Lee et al., 2025), dynamic endothelial cells (ECs) rapidly switch from a quiescent to a highly proliferative state by increasing their biosynthetic and bioenergetic demands (Adams and Alitalo, 2007). Although ECs are highly dependent on glycolysis for proliferation and migration during angiogenesis, accumulating evidence has shown that EC mitochondria play a crucial role in angiogenesis, particularly in disease states by functioning as signaling organelles and supplying the biosynthetic molecules necessary for EC growth (Schiffmann et al., 2020; Wang et al., 2022; Luo et al., 2023). Angiogenic stimuli by VEGF were reported to increase mitochondrial oxidative respiration and ATP levels in ECs (Zimbone et al., 2025). The mitochondrial protein FAM3A increases capillary density and angiogenesis via activation of VEGFA transcription (Xu et al., 2019). Several resident mitochondrial proteins, such as TIMM (Ma et al., 2023), SIRT3 (Jiang et al., 2025), and WARS2 (Hu et al., 2025), have been reported to be associated with angiogenesis. Mitochondrial membrane proteins, along with mitochondria-associated membranes (MAMs), are recognized as other key factors in the pathophysiological process of angiogenesis since the mitochondrial membrane is a central sensor for signal transduction and for mitochondrial morphology and functional maintenance (Favia et al., 2014; Gӧbel et al., 2020). Optic atrophy proteins, primarily OPA1, are upregulated in response to VEGF stimuli and are required for tumor vascularization via the NF-κB pathway (Herkenne et al., 2020). Silencing FUN14 domain-containing 1 (FUNDC1) inhibits tumor angiogenesis by decreasing the formation of MAMs (Wang et al., 2021). Lack of MFN1 leads to EC dysfunction (Gӧbel et al., 2020), and MFN2 in astrocytes prevents injury-induced vascular remodeling (Lugus et al., 2011). Additionally, SLC25A6/ANT3, a membrane protein involved in ADP/ATP antiporter activity, is suggested to mediate mitochondrial membrane permeability, which triggers apoptosis or death via interaction with CypD (Wu et al., 2020), whereas the deletion of CypD promotes VEGF-induced proliferation and angiogenesis (Marcu et al., 2015). However, our understanding of the mechanism through which mitochondria regulate EC angiogenesis remains elusive.

X-prolyl aminopeptidase 2 (XPNPEP2), a membrane protein that has the catalytic activity of removal of a penultimate prolyl residue from the N-termini of peptides, is abundantly expressed in vascular ECs and epithelial cells in the intestine and renal proximal tubule (Simmons and Orawski, 1992; Erşahin and Simmons, 1997; Matsui et al., 2003). Recent genetic studies have suggested that the *XPNPEP2* gene may contribute to the development of angiotensin-converting enzyme inhibitor (ACEi)-associated angioedema, which is characterized by injured ECs, abnormal vascular permeability, and inflammation (Matsui et al., 2003; Cilia La Corte et al., 2011). Decreased expression and activity of *XPNPEP2* have been reported in hypertensive patients (Cilia La Corte et al., 2011), whereas overexpression of *XPNPEP2* has been reported in patients with cervical cancer and clear-cell renal cell carcinoma (Cheng et al., 2017; Wang et al., 2020). Moreover, recent studies found that XPNPEP2 was a potential risk factor in premature ovarian insufficiency (Illés et al., 2024) and was involved in heatstroke-induced coagulopathy due to vascular endothelial damage (He et al., 2025). All indications suggested that XPNPEP2 plays a role in EC function and vascular biology, although the underlying mechanism remains largely unknown.

In this study, our results revealed that XPNPEP2 is involved in EC angiogenesis and is essential for EC function in an Xpnpep2 deletion mouse model and XPNPEP2-knockdown human umbilical vein endothelial cells (HUVECs). XPNPEP2 interacts with SLC25A6, and XPNPEP2 deficiency promotes Siah E3 ubiquitin protein ligase 1 (SIAH1)-mediated proteasomal degradation of SLC25A6, leading to mitochondrial dysfunction and consequently inhibiting angiogenesis.

## 2 Materials and methods

### 2.1 Mouse models

*Xpnpep2* ^*KO*^ mice were generated using the CRISPR/Cas9 system, targeting genomic RNA GGAATCTCTCTGCCTACATC for *Xpnpep2* by Biogle (Jiangsu, China). Mice genotypes were confirmed via PCR amplification and Sanger sequencing using the following primers: forward, CATCCCATCACTCTTAAATATAGC; reverse, TGTCTTCAGCTTCCACATCTTAC. The mouse bearing 52 bp deletion with 2 bp insertion in exon 3 was selected. The information on *Xpnpep2* ^*KO*^ is listed in Supplementary Figure S2. All animal protocols were approved by the Animal Care and Use Committee of the Zhejiang University School of Medicine.

### 2.2 Cell culture and plasmids

All cells were grown at 37 °C in an incubator at 5% CO_2_. HUVECs between passages 4 and 10 were used and cultured in EGM-2 BulletKit medium (Lonza) supplemented with 5% fetal bovine serum (FBS), with or without VEGF (50 ng/mL, MCE). HEK293T and mouse Lewis lung cancer (LLC) were cultured in DMEM supplemented with 10% FBS. The PLKO.1 plasmid carrying short hairpin RNA (shRNA) oligos was packaged into lentiviruses by co-transfecting HEK293T cells with a plasmid mixture (shRNA, psPAX2, and pMD2.G packaged at 4:3:1). The viral supernatant was collected at 48 h and 72 h post-transfection, filtered through a 0.45-μm membrane, and stored for subsequent use. HUVECs were then transduced with this viral supernatant to establish the XPNPEP2-, SLC25A6-, or SIAH1-knockdown cell line. XPNPEP2 or SLC25A6 cDNA was constructed into the PLVX-puro vector and packaged into lentiviruses. The pcDNA3.1(+)-FLAG and pcDNA3.1(+)-HA vectors were used to overexpress cDNA of XPNPEP2/SLC25A6 (with silent mutations in the shRNA target sequence) using Hieff Trans™ liposomal transfection reagent according to the manufacturer’s protocol. The primers of shRNA and scramble are listed in Supplementary Table S1.

### 2.3 Co-immunoprecipitation (Co-IP) and Western blotting (WB)

For co-immunoprecipitation, HEK293T was collected and lysed in IP lysis buffer containing PMSF (protease inhibitor) on ice for 20 min and then centrifuged. The supernatant was incubated with 20 μL protein A/G plus agarose beads (Santa Cruz Biotechnology, Shanghai, China) and 1.0 μg IgG at 4 °C for 30 min. After centrifugation at 5,000 rpm, pretreated cell lysate was transferred to a new EP tube and incubated overnight with primary antibodies. Next day, samples were incubated with 25 μL beads overnight at 4 °C. Last day, agarose beads were washed and eluted with lysis buffer at 98 °C for 10 min. Then 20 μL input and IP samples were subjected to Western blot assay.

For whole-cell lysates, 25 μg protein per lane was denatured and electrophoresed on an 8%–10% SDS-PAGE gel and then transferred onto a polyvinylidene difluoride (PVDF) membrane. The membranes were blocked in 5% (w/v) milk for 2 h at room temperature and incubated with primary antibodies overnight at 4 °C. HRP-conjugated anti-rabbit or anti-mouse IgG was used as secondary antibodies. Signals were detected using ECL kits (Vazyme, China) and visualized using a Clinx-Chemi-Capture system. Primary antibodies are listed in Supplementary Table S2.

### 2.4 Immunofluorescence (IF) staining

HEK293T and HUVECs seeded on coverslips were incubated with 100 nM MitoTracker at 37 °C for 20 min, then fixed in 4% paraformaldehyde with 0.1 M phosphate buffer (PFA) for 20 min, permeabilized in 0.3% Triton X-100 for 15 min, blocked in 3%–5% bovine serum albumin (BSA) for 1 h at room temperature, and then incubated with primary antibodies overnight at 4 °C. The next day, cells were rinsed, co-incubated with fluorescent secondary antibodies, Alexa Fluor 488 or 568, for 1 h, and finally counterstained with DAPI for 10 min. Images were captured using ZEISS LSM 880 and analyzed using ImageJ. Antibodies are listed in Supplementary Table S2.

### 2.5 Immunohistochemistry

Mouse organs and tissue were excised and fixed in 4% PFA over 24 h at room temperature to make paraffin cubes and sections. Sections were dewaxed and de-benzenized. Afterward, antigen retrieval was performed. Sections were blocked in 3% FBS for 1 h, subsequently stained with primary antibodies for 1 h at room temperature, followed by incubation with second antibodies for 15 min. Finally, the sections were stained with hematoxylin and eosin, and then images were captured using a Nikon eclipse 80i microscope with ImageJ analysis.

### 2.6 XPNPEP2 enzyme activity assay

K(Dnp)PPK(Abz)NH2, containing the fluorescent group Abz (o-aminobenzoicacid) and the quencher Dnp (2,4-dinitrophenyl), was used as substrate to detect enzyme activity. A substrate solution [1.5 μmol/L substrate, 0.5 mmol/L MnCl_2_, and 0.1 mol/L HEPES (pH 8.0)] was added into the 96-well plate containing cell lysate and then incubated in a microplate reader at 37 °C for 3–3.5 h to record the linear change in fluorescence.

### 2.7 Mouse fluorescein angiography

Fluorescent angiography of eyes was carried out using the Micron III camera (Phoenix Research Laboratories, Inc., Pleasanton) as described previously (Spaide et al., 2015; Yu et al., 2020). Pupils were dilated with 1% tropicamide (Bausch & Lomb, Tampa). Systane lubricant eye drops (Alcon) were used to maintain the cornea moist. Pupils were intraperitoneally injected with 0.2% fluorescein sodium (Alliance Pharmaceutical, Inc., San Diego) at a dose of 0.01 mL/g of body weight, and subsequently, images were captured.

### 2.8 Mouse retinal vessel labeling

The whole retinas were fixed with 4% PFA for 1 h and then blocked with 3% FBS and 0.3% Triton X-100 at 4 °C overnight. Next day, retinas were equilibrated with the solution (1 μM MgCl_2_, 1 μM CaCl_2_, 0.1 μM MnCl_2_, and 0.1% Triton X-100 in PBS) for 1 h at room temperature. After incubating with FITC-conjugated isolectin B4 (Sigma-Aldrich, St. Louis) at 4 °C overnight, the retinas were washed, flat-mounted, and then imaged using Leica DM4000 B LED. The images were analyzed using ImageJ and AngioTool.

### 2.9 Mouse aortic ring assay

Pulmonary arteries were removed from mice (at 8 weeks) and cut into 1-mm rings as reported previously (Baker et al., 2011). In brief, each aortic ring was placed in a 96-well plate containing 100 μL growth factor-reduced Matrigel (Corning, Shanghai, China) per well. Then the rings were incubated in culture medium (opti-MEM, 1% penicillin/streptomycin, 50 ng/mL VEGF, and 2.5% FBS). The EGM-2 medium was changed initially on day 3 or day 4 and then every other day until the end of the experiments. The sprouting area was recorded under a phase-contrast microscope (Leica Microsystems) on day 7 and analyzed using ImageJ.

### 2.10 Mouse cutaneous wound-healing experiments

Wound-healing experiments were performed, as previously described (Willenborg et al., 2012). In brief, 1-cm^2^ square-biopsy wounds were induced on the shaved back of *Xpnpep2* ^*KO*^ and WT mice at 8 weeks. The healing rate was calculated by measuring the area of wound on 0, 3, and 7 days. The full-layer wound skins on 7 days were removed to perform immunohistochemistry assay.

### 2.11 Mouse subcutaneous tumor models

A total of 4 × 10^6^ cells in 100 μL PBS were subcutaneously implanted in the dorsal flank of 8–12-week-old mice, as previously described (Xu et al., 2021). The tumor volumes were measured using a digital caliper every other day and calculated using the formula V = 0.52 × L × W^2^. Fifteen days after planting, tumors were fixed with 4% PFA for immunohistochemistry assay.

### 2.12 In-gel activity assay

For detecting the activities of OXPHOS complexes I, II, IV, and V in gel, 30 μg mitochondrial protein from tissues was subjected to 3%–11% gradient Bis-Tris NativePAGE gel and run at a constant voltage of 150 V in dark blue buffer for 1 h and then at 250 V in light blue cathode buffer at 4 °C for 2 h. The native gels were prewashed in cold water and then incubated with the corresponding fresh substrate of complex I [0.1 mg/mL NADH, 2.5 mg/mL nitrotetrazolium blue chloride (NTB), and 2 mM Tris-HCl, pH 7.4], complex II (1 M sodium succinate, 2.5 mg/mL NTB, 0.2 mM phenazine methosulfate, and 5 mM Tris-HCl, pH 7.4), complex IV [0.5 mg/mL diaminobenzidine (DAB), 1 mg/mL cytochrome c, and 45 mM phosphate buffer, pH 7.4], and complex V [35 mM Tris-HCl pH 7.4; 14 mM MgSO_4_; 270 mM glycine; 10 mM ATP; and 0.2% Pb(NO_3_)_2_] overnight at 4 °C. Enzymatic reactions were stopped using 10% acetic acid. The gels were washed and then scanned for the evaluation of enzymatic activity.

### 2.13 Subcellular fractionation and localization

HUVECs were collected and homogenized on ice in isolation buffer 2 (225 mM mannitol, 75 mM sucrose, and 30 mM Tris-HCl). The supernatant (containing endoplasmic reticulum and lysosomes) and precipitates (crude mitochondria) were separated through centrifugation. The supernatant was separated into cytoplasm (upper) and endoplasmic reticulum (lower) through ultracentrifugation (4 °C, 100,000 × g, 1 h, Beckman Coulter Optima L-80XP). Purified mitochondria and MAMs were isolated from cells derived from 10 culture dishes through ultracentrifugation combined with Percoll density gradient centrifugation as previously described (Kristian, 2010; Yu et al., 2020). In brief, Percoll medium, crude mitochondria, and MRB buffer were gradually added to ultracentrifuge tubes, respectively, and centrifuged at 4 °C and 95,000 × g for 30 min to separate mitochondria and MAM layers (de Brito and Scorrano, 2008). Proteins from each cellular fraction were prepared for subsequent Western blot assay.

### 2.14 Transmission electron microscopy

Mitochondrial morphology of HUVECs was examined using transmission electron microscopy (TEM). Cells were fixed in 2.5% glutaraldehyde for 24 h, post-fixed in 1% OsO_4_, dehydrated in a graded series of ethanol solution, washed in acetone, and embedded in resin mixture at room temperature overnight. Embedded samples were sliced using an EM UC7 ultratome (Leica, Germany) and stained with uranyl acetate and lead citrate for 15 min. Images were obtained using an H-7650 transmission electron microscope (Hitachi, Japan).

### 2.15 Scratch wound migration assay

HUVECs were seeded in 24-well plates. After the cells spread evenly over the bottom surface of plates, a scratch was made using a 200-μL pipette tip. Migration of the cells into the wound was recorded 12 h later and analyzed using ImageJ.

### 2.16 Tube formation assay

HUVECs were seeded in the 24-well plates, which were pretreated with Matrigel (150 μL per well) at a 40% density per well and incubated at 37 °C for 12 h. The sprouting of ECs to form tubes was recorded and acquired using a fluorescence microscope (Leica DM4000 B LED). The number of branch points and tube length were quantified using ImageJ.

### 2.17 Measurements of oxygen consumption and extracellular acidification

The oxygen consumption rate (OCR) and extracellular acidification rate (ECAR) in HUVECs were measured using an XF-96 extracellular flux analyzer (Seahorse Bioscience), as detailed elsewhere (Yu et al., 2020). The OCR was measured after the sequential addition of oligomycin (1 μM), FCCP (2.5 μM), antimycin A (5 μM), and rotenone (1 μM). The ECAR was measured after the addition of glucose (10 mM), oligomycin (1 μM), and 2-DG (50 mM) using a procedure similar to that used for OCR.

### 2.18 ATP measurements

Mitochondrial ATP levels of cells were measured using the CellTiter-Glo luminescent cell viability assay (Promega, G7571, Madison, WI, United States). According to the manufacturer’s instructions, 1 × 10^4^ cells/well were seeded in plates, and 100 μL of substrate-mixed buffer was added on a shaker to induce cell lysis. After incubation at room temperature for 10 min, the luminescence was detected using a microplate reader (Thermo Fisher Scientific, Waltham, MA, United States).

### 2.19 mtDNA copy-number analysis

To extract the total DNA from the cells, the total DNA extraction kit (TIANGEN, Cat. No. DP304. Beijing, China) was used. Quantitative real-time PCR (qPCR) was performed to measure the mtDNA copy number using SYBR Green dye. *β-Actin* and *mt-ND1* were used as target genes to calculate the mtDNA relative quantification.

### 2.20 Quantification and statistical analysis

Data were analyzed using GraphPad Prism 8.0. and presented as mean ± SD of triplicates. Comparisons between two groups were performed using two-tailed Student’s t-test. A *p-*value <0.05 was considered statistically significant.

## 3 Results

### 3.1 XPNPEP2 regulates angiogenesis *in vivo*

**FIGURE 1:**
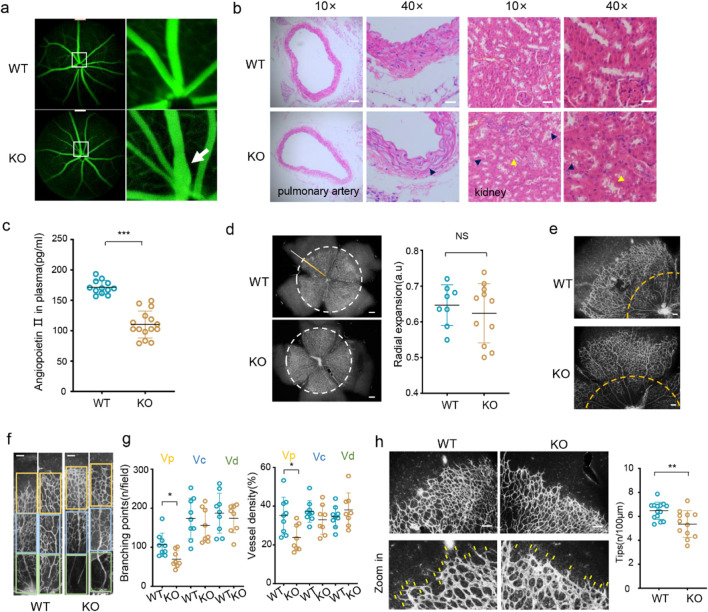
XPNPEP2 deletion disrupts vascular function and angiogenesis *in vivo*. **(a)** Mouse fluorescein retinal angiography. The arrow indicates the abnormal area. **(b)** HE staining of pulmonary artery and kidney sections from *Xpnpep2* ^WT^ and *Xpnpep2* ^KO^ mice; scale bar = 100 μm (×10); 25 μm (×40). Black arrowheads indicate renal tubular vacuolation, and yellow arrowheads indicate epithelial cell exfoliation (n = 4/genotype, triplicate experiments). **(c)** Detection of angiotensin II in mouse plasma. **(d–h)** Isolectin b4 immunofluorescence of flat-mounted retinas from 5-day-old pups of the indicated genotype (n > 5/genotype). **(d)** Radial expansion of mouse retinal blood vessels and the quantification of radial expansion (vessel extension radius/retinal radius); scale bar = 200 μm. **(e)** Branching and density of retinal veins. The yellow dotted line indicates the vascular proximal region; scale bar = 100 μm. **(f)** Branching and density of venous vessels in proximal (Vp), middle (Vc), and distal (Vd) regions; scale bar = 100 μm. **(g)** Quantification of branching points and density. **(h)** Tips showing the numbers of vessels in the distal region; scale bar = 100 μm. Yellow rods indicate vessel sprouting. Data represent mean ± SD of triplicates. *, *P* < 0.05; **, *P* < 0.01; ***, *P* < 0.001; NS, no significance.

The public database of Gene Expression Omnibus (GEO) profiles revealed that lower levels of *XPNPEP2* are present in failing heart or hypertensive mouse artery, hypertensive nephropathy, and anti-angiogenesis models (Supplementary Figure S1a), indicating its potential role in EC function. Transcriptional changes in *XPNPEP2* have also been identified in response to VEGF stimulation in HUVECs in the available ANGIOGENES database (http://angiogenes.uni-frankfurt.de). Additionally, increased protein levels of XPNPEP2 were confirmed upon VEGF treatment for 1–2 h *in vitro* (Supplementary Figure S1b). These indications suggested that XPNPEP2 may contribute to abnormal EC function and angiogenesis under certain conditions. To clarify whether XPNPEP2 defects lead to vascular abnormalities *in vivo*, we generated an *Xpnpep2*-knockout mouse model (*KO*: *Xpnpep2* ^*−/−*^ or *Xpnpep2* ^*-/Y*^) using the CRISPR/Cas9 system and verified its genotype using Sanger sequencing (Supplementary Figures S2a–c). The level of the XPNPEP2 protein was apparently diminished, as shown by WB and IF in retina and kidney tissues from homozygous mice, without alterations in body weight in those under 20 weeks of age (Supplementary Figures S2d–f). First, we observed the retinal vascular cells through angiography and found that two-thirds of the KO mice (8/12) had tortuous and dilated lumens in their retinal vascular cells compared with those in the WT mice (Figure 1a). The tortuosity of vasculature is based on expansion due to hardening and reduced elasticity of the vessel wall combined with the long-term impact of blood flow (Davutoglu et al., 2013). Moreover, we dissected the mouse pulmonary arterial rings and kidney tissues and fixed them for hematoxylin–eosin (HE) staining. The arterial walls showed relaxation of the external elastic fibers, whereas renal tissues exhibited vacuolar degeneration and exfoliation, consistent with ischemic injury (Figure 1b). A significant decrease in the concentration of plasma angiotensin II (Ang II) measured in blood plasma was detected in the KO mice (110 pg/mL) compared with the WT mice (171 pg/mL) (Figure 1c). These data indicate that XPNPEP2 defects induce pathological changes in the vasculature.

To explore whether ablation of XPNPEP2 affects the formation of blood vessels, we used a mouse retina model of physiological angiogenesis. The mouse retina is avascular at birth, and blood vessels gradually develop from the center to the periphery until postnatal day 7. We dissected the retinas of 5-day-old pups and used the EC marker isolectin B4 to stain retinal vessels to observe angiogenesis in the mice. No discernible variation in the radial expansion of the retinal vascular plexus or the general growth of the arterial branches was observed between the WT and KO mice (Figures 1d,e; Supplementary Figure S3a). However, we divided the veins into three areas, proximal, middle, and distal, and counted the vascular density and number of branch points. The proximal vessels in the KO mice presented notable decreases in vessel density (65% of those in the WT mice) and branch points (68% of those in the WT mice) (Figures 1f,g). Moreover, the loss of Xpnpep2 decreased the number of tips, which spread filopodia during vessel growth to mirror physiological extension (Figure 1h). These findings suggest that the loss of XPNPEP2 disturbs some physiological processes involved in angiogenesis.

**FIGURE 2:**
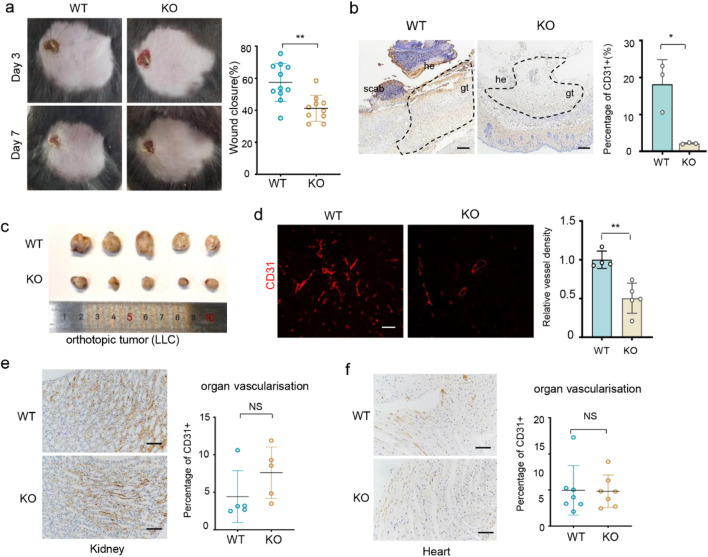
Xpnpep2 deletion prevents neoangiogenesis in wound healing and tumor growth. **(a)** Macroscopic wound images of *Xpnpep2* ^WT^ and *Xpnpep2* ^KO^ mice and the quantification of wound closure at day 7 post-injury (n = 11 vs. 10). **(b)** CD31 staining of wound sections derived from mice of each genotype at day 7 post-injury and the quantification of CD31^+^ proportion in granulation tissue. gt, granulation tissue; he, hyperproliferative epithelium; scale bar = 100 μm (n ≥ 3/genotype, triplicate experiments). **(c)** Images of explant LLC tumors from *Xpnpep2* ^KO^ and *Xpnpep2* ^WT^ mice. **(d)** Immunofluorescence images of CD31 in LLC tumors from *Xpnpep2* ^KO^ and *Xpnpep2* ^WT^ mice (n = 5/genotype) and the quantitative data of relative vessel density; scale bar = 50 μm. **(e,f)** CD31 staining of the Kidney **(e)** and Heart **(f)** sections derived from *Xpnpep2* ^KO^ and *Xpnpep2* ^WT^ adult mice and the quantification of CD31^+^ proportion in granulation tissue (n ≥ 3/genotype, triplicate experiments); scale bar: 25 μm. Data represent mean ± SD of triplicates. *, *P* < 0.05; **, *P* < 0.01; ***, *P* < 0.001; NS, no significance.

ECs have an approximately quiescent renewal rate of several months in healthy adults (Ricard et al., 2021) and dramatically increase their proliferation rate under disease stress. To demonstrate that XPNPEP2 ablation prevents neoangiogenesis under disease conditions, we constructed a mouse model of skin incision wound healing. Full-thickness excisional cube wounds were made on the backs of WT and KO mice. As shown in Figure 2a, wound closure was significantly delayed in the KO mice compared with the control mice. CD31 [platelet EC adhesion molecule 1 (PECAM-1)] reliably marks both mature endothelium and nascent capillaries. Histological analysis revealed that the vascularization of granulation tissue was disrupted with a lower number of CD31^+^ cells, from a mean value of 18% in WT to 2% in KO (Figure 2b). To confirm the role of XPNPEP2 in neoangiogenesis, we generated an orthotopic tumor model by subcutaneous implantation of LLC cells into C57BL/6 mice. Compared with that in WT mice, tumor growth in KO mice was noticeably suppressed (Figure 2c; Supplementary Figures S3b,c). The percentage of CD31^+^ tumor vessels in KO mice was markedly lower (48%) than that in the controls (Figure 2d). However, the coverage rate of CD31^+^ cells in existing vessels was not significantly different between WT and KO mice according to histological analysis of either kidney or heart tissues (Figures 2e,f). These data revealed that XPNPEP2 deletion prevented injury-induced neoangiogenesis and had no effect on quiescent vasculature under homeostatic conditions.

### 3.2 XPNPEP2 is essential for EC function

**FIGURE 3:**
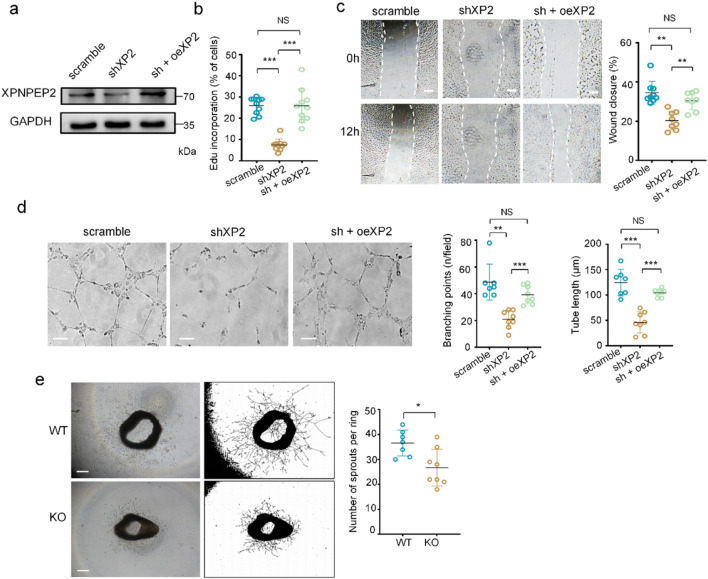
XPNPEP2 is essential for EC function. **(a)** Western blot analysis of XPNPEP2 expression in shXPNPEP2 and control HUVECs. GAPDH was used as the loading control. **(b)** HUVEC proliferation using Edu incorporation. **(c)** Scratch wound assay of HUVECs and the quantification of wound closure; scale bar = 250 μm. **(d)** Tube formation of HUVECs and the quantification of branching points and tube length; scale bar = 100 μm. **(e)** Bright-field images of pulmonary aortic rings at day 8 post-culturing in Matrigel from *Xpnpep2* ^WT^ and *Xpnpep2* ^KO^ mice and the quantitative statistics of sprouts per ring; scale bar = 500 μm (n ≥ 7/genotype). shXP2, XPNPEP2 with shRNA interference; oeXP2, XPNPEP2 overexpression (with silent mutations in the shXP2 target sequence). Data represent mean ± SD of triplicates. *, *P* < 0.05; **, *P* < 0.01; ***, *P* < 0.001; NS, no significance.

EC proliferation and migration initiate the formation of capillary networks. To investigate whether XPNPEP2 is required for angiogenesis *in vitro*, we efficiently silenced XPNPEP2 in HUVECs using lentivirus-delivered shRNA (Supplementary Figure S4a). Given the crucial role of mitochondria in EC function, we first assessed whether XPNPEP2 deletion altered mitochondrial content by measuring the mtDNA copy number, a key indicator of mitochondrial biogenesis. Inhibition of XPNPEP2 had no effect on the mtDNA copy number (Supplementary Figure S4b), indicating that the mitochondrial impairment was not due to changes in mitochondrial mass but likely in mitochondrial function. However, HUVEC proliferation and migration were clearly inhibited, as indicated by the results of the EdU-based cell proliferation (Supplementary Figure S4c) and scratch wound migration assays (Supplementary Figure S4d). Furthermore, we expressed XPNPEP2 (oeXP2) into XP2-knocked down cells (shXP2) to determine whether EC function was restored (Figure 3a). XPNPEP2 clearly increased the number of proliferated cells from a mean value of 7% in the shXP2 group to 25% in the control group (Figure 3b). Consistent with the findings in the KO mice, the wound closure capacity was significantly diminished when XPNPEP2 was absent in the HUVECs but recovered from 20% to 30% when XPNPEP2 expression was restored, which was slightly lower than that of the controls (34%) (Figure 3c). Impaired tubulogenesis in the formation of a capillary network is usually characterized by a decrease in the number of branch points and tube length. As shown in Figure 3d, both branch points and tube length were dampened without XPNPEP2, which was effectively reversed by exogenous XPNPEP2 expression. Aortas from WT and KO mice were also evaluated for *ex vivo* vascular remodeling using an aortic ring angiogenesis assay. Compared with their WT counterparts, XPNPEP2^KO^ aortic rings presented significantly reduced aortic ring sprout formation in response to VEGF stimulation (Figure 3e). Taken together, these results suggest that XPNPEP2 is essential for EC function and that its deficiency impairs EC function and angiogenic capacity.

### 3.3 Silencing XPNPEP2 leads to mitochondrial dysfunction

**FIGURE 4:**
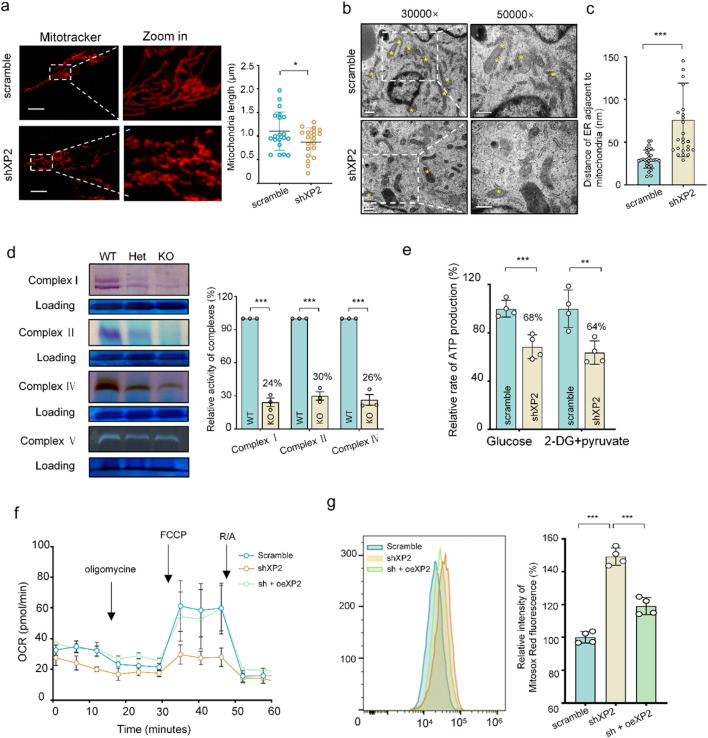
Silencing XPNPEP2 altered mitochondrial morphology and function. **(a)** Mitochondria morphology analysis with MitoTracker^®^ staining in HUVECs and relative lengths of mitochondria; scale bar = 10 μm. **(b)** Mitochondria and MAM morphology using TEM in HUVECs. Yellow asterisks indicate MAMs; scale bar = 0.5 μm. **(c)** Quantitative analysis of the distance of ER adjacent to mitochondria. **(d)** In-gel activity of complexes I, II, IV, and V in the mouse liver using NativePAGE and quantitative analysis of the relative activities of complexes. Coomassie staining was used as the loading control. **(e)** Measurement of ATP levels in HUVECs. Group glucose represents the total ATP level, and group 2-DG + pyruvate represents the ATP level of OXPHOS (n = 4). **(f)** The rates of OCR (O_2_ consumption) in HUVECs when XPNPEP2 is absent and rescued. Oligomycin, FCCP, and rotenone and antimycin A (R/A) were used as inhibitors. **(g)** Mitochondrial ROS in HUVECs, measured using flow cytometry with MitoSOX (n = 3). shXP2, XPNPEP2 with shRNA interference; oeXP2, XPNPEP2 overexpression (with silent mutations in the shXP2 target sequence). Data represent mean ± SD of triplicates. *, *P* < 0.05; **, *P* < 0.01; ***, *P* < 0.001.

Considering that mitochondrial dynamics and respiration contribute to angiogenesis (Cannito et al., 2025), we assumed that whether XPNPEP2 plays a role in angiogenesis by modulating mitochondrial function. Here, we observed mitochondrial fragmentation in HUVECs lacking XPNPEP2 (Figure 4a). These results were also observed in TEM images. In addition, the number of MAMs, which play an important role in maintaining intracellular homeostasis and mitochondrial functions (Yang et al., 2020), was reduced, and their distance (between mitochondria and adjacent ER) was increased to a mean value of 75 nm in shXP2 HUVECs compared with that of 30 nm in controls (Figures 4b,c), suggesting the destruction of MAMs, at least in part. Therefore, we assessed the complex activity of the mitochondrial respiratory chain using in-gel activity assays as described previously (Tong et al., 2023). The activities of complexes I, II, and IV were drastically decreased in liver tissue from KO mice at levels of 24%, 30%, and 26% compared to those in tissues from WT mice, respectively (Figure 4d). Consistently, mitochondrial ATP production was dampened in shXP2 HUVECs, with a mean value of 64% relative to that of the controls (Figure 4e). We further evaluated the OCRs of HUVECs to assess respiratory capacity in the absence of XPNPEP2 (Xu et al., 2022). As shown in Figure 4f, the OCR was significantly decreased in HUVECs without XPNPEP2, whereas overexpressing XPNPEP2 into shXP2 cells rescued mitochondrial respiratory chain function. Moreover, the production of mitochondrial reactive oxygen species (mROS) was noticeably increased to 150% in shXP2 HUVECs and to 119% when XPNPEP2 was expressed in shXP2 HUVECs, as measured using a MitoSOX indicator (Figure 4g). As expected, vacuolar and fragmented mitochondria were reduced when XPNPEP2 was expressed into shXP2 cells (Supplementary Figure S5a). However, glycolysis is described as a necessity for EC metabolism and angiogenesis. To exclude the impact of glycolysis on EC function with or without XPNPEP2, we carried out glycolytic capacity analysis of HUVECs using the ECAR assay. No significant differences were detected in ECAR values or in the expression of glycolysis-related proteins, GPI and ENO1, between the shXP2 HUVECs and the controls (Supplementary Figures S5b–d). Together, our data revealed that XPNPEP2 deletion caused mitochondrial dysfunction without significant glycolytic interference.

### 3.4 XPNPEP2 interacts with SLC25A6

**FIGURE 5:**
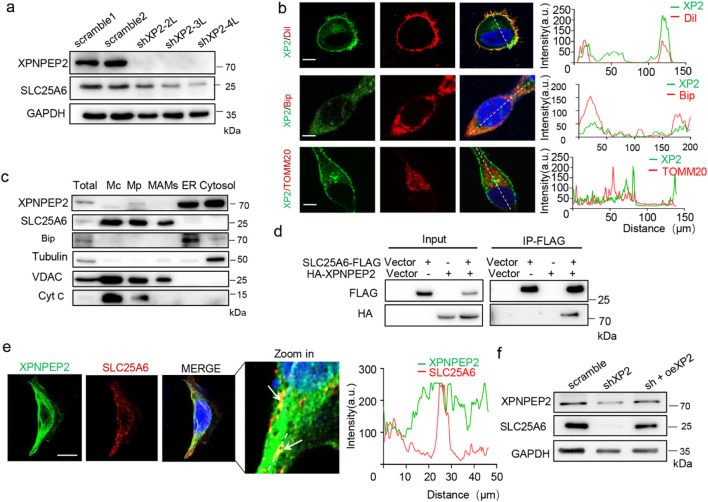
XPNPEP2 interacts with SLC25A6. **(a)** SLC25A6 expression in shXP2 and control HUVECs. GAPDH was used as the loading control. **(b)** Immunofluorescence staining for the subcellular localization of XPNPEP2 in HEK293T; n = 3 independent experiments; Dil (plasma membrane marker), Bip (ER marker), and TOMM20 (mitochondrial marker); scale bar = 50 μm. **(c)** Subcellular localization of XPNPEP2 and SLC25A6 in HEK293T. Bip (ER marker), tubulin (cytosol marker), VDAC (OMM and MAM marker), and Cyt C (mitochondrial matrix marker). Mc, crude mitochondrial fraction; Mp, purified mitochondria. **(d)** Co-immunoprecipitation of SLC25A6-FLAG with HA-XPNPEP2 in HEK293T. **(e)** Co-localization of XPNPEP2 and SLC25A6 in HEK293T; scale bar: 10 μm. **(f)** Overexpression of XPNPEP2 rescued SLC25A6 defect in shXP2 HUVECs. GAPDH was used as the loading control.

To validate how XPNPEP2 modulates mitochondrial function in angiogenesis, we extracted RNA sequencing (RNA-seq) data from the ANGIOGENES database and intersected the list of differentially expressed genes with the Human MitoCarta 3.0 database (Rath et al., 2021). We identified 804 mitochondrial genes whose transcription dynamically changed during VEGF stimulation of HUVECs (Supplementary Table S3). Some genes associated with mitochondrial dynamics, such as *SLC25A6*, *MFN1*, *MFN2*, *MFF*, *DNM1L*, and *FIS1*, were distinctly upregulated 4 h after stimulation compared with the 1-h data (Supplementary Figure S6a), suggesting their correlation with angiogenesis. Western blotting revealed that a reduction in XPNPEP2 expression was concomitant with a dramatic decrease in SLC25A6 expression (Figure 5a; Supplementary Figure S6b). Moreover, we analyzed the protein profile of immunoprecipitates enriched with HA-tagged XPNPEP2 using liquid chromatography–tandem mass spectrometry and identified SLC25A6 proteins that overlapped (Supplementary Table S4), suggesting that SLC25A6 potentially interacts with XPNPEP2. We then examined the subcellular distributions of XPNPEP2 and SLC25A6 to explore their implications in MAMs. Immunofluorescence experiments revealed that XPNPEP2 was localized mainly at the cyto-membrane and endoplasmic reticulum (ER) in HEK293T (Figure 5b). To verify their localization in detail, we performed subcellular fractionation analysis of XPNPEP2 and SLC25A6 through density gradient centrifugation and found that SLC25A6, like VDAC, was enriched in MAMs and mitochondria, whereas XPNPEP2 was enriched in the ER and cytosol (Figure 5c), suggesting the possibility of spatial interactions between them. Co-immunoprecipitation analysis of HEK293T cells revealed the physical interaction between XPNPEP2 and SLC25A6 (Figure 5d; Supplementary Figure S6c). Accordingly, the co-localizations of XPNPEP2 and SLC25A6 were visualized using immunofluorescence in HEK293T cells (Figure 5e). The Pearson’s correlation coefficient was calculated from a total of more than 90 cells across three independent replicates (n ≥ 30 cells per replicate), yielding a value of 0.668, indicating a moderate-to-strong positive correlation between XPNPEP2 and SLC25A6. Additionally, SLC25A6 downregulation caused by XPNPEP2 knockout was reversed by the overexpression of exogenous XPNPEP2 (Figure 5f). In contrast, silencing SLC25A6 in HUVECs resulted in a significant reduction in XPNPEP2 (Supplementary Figure S6d). Taken together, our findings here revealed that XPNPEP2 interacted with SLC25A6 and was required for the steady-state level of SLC25A6.

### 3.5 XPNPEP2 ablation increases SLC25A6 ubiquitination via SIAH1

**FIGURE 6:**
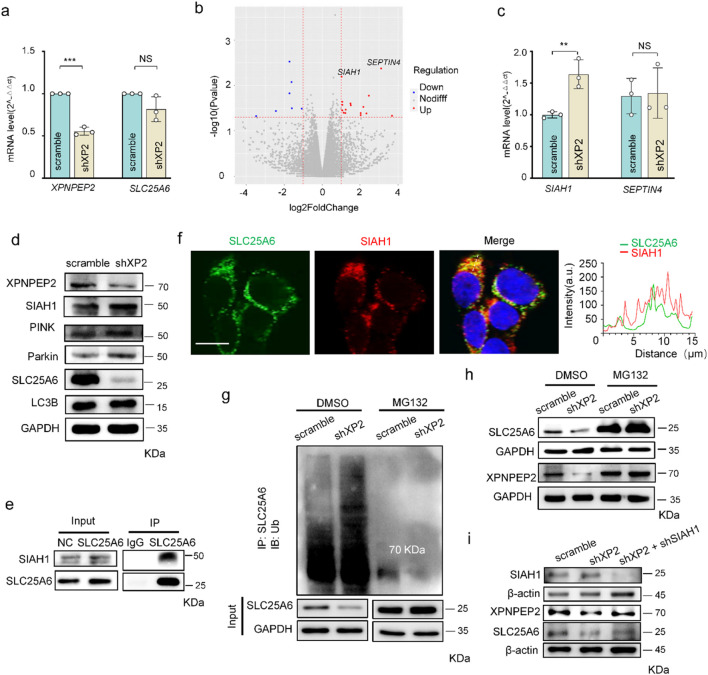
XPNPEP2 ablation increased the ubiquitination of SLC25A6 via SIAH1. **(a)** mRNA expression of XPNPEP2 and SLC25A6 in shXPNPEP2 and control HUVECs. **(b)** Volcano plot of differentially expressed genes in shXPNPEP2 and control HUVECs. Red and blue dots correspond to genes upregulated and downregulated, respectively; n = 3 independent experiments. **(c)** mRNA expression of SIAH1 and SEPTIN4 in shXPNPEP2 and control HUVECs. **(d)** Western blot analysis of SIAH1 (Proteintech 13886-1-AP), SEPTIN4, and mitophagy-associated proteins in shXPNPEP2 and control HUVECs. GAPDH was used as the loading control. **(e)** Physical interaction of SIAH1 with SLC25A6, using anti-SLC25A6 antibodies for IP. **(f)** Co-localization of SLC25A6 and SIAH1 by immunofluorescence in HEK293T; scale bar = 20 μm. **(g)** SLC25A6 ubiquitination in shXPNPEP2 and control HUVECs, with or without MG132 treatment. GAPDH was used as the loading control. **(h)** Western blot analysis of XPNPEP2 and SLC25A6 in shXPNPEP2 and control HUVECs, with or without MG132 treatment. GAPDH was used as the loading control. **(i)** SLC25A6 expression in HUVECs with or without SIAH1 (Thermo PA5-88583) when XPNPEP2 is absent. β-Actin was used as the loading control. Data represent mean ± SD of triplicates. **, *P* < 0.01; ***, *P* < 0.001; NS, no significance.

To determine how SLC25A6 deficiency occurs when XPNPEP2 is lost, we examined SLC25A6 mRNA transcription in controls and shXP2 HUVECs. No marked reduction in *SLC25A6* mRNA levels was observed between the control and shXP2 groups (Figure 6a). Given the interaction between XPNPEP2 and SLC25A6, we considered that this interaction may be involved in protein degradation of SLC25A6 triggered by XPNPEP2. We therefore compared the RNA-seq data between the control and the shXP2 groups, in which 23 genes were differentially expressed (Supplementary Table S5). Among them, *SIAH1* and *SEPTIN4*, genes involved in ubiquitination, were particularly upregulated (Figure 6b) as ubiquitination is important for protein degradation (Zhang et al., 2020; Zhou et al., 2024). The qPCR experiments confirmed that only *SIAH1* mRNA was indeed upregulated without XPNPEP2 (Figure 6c). Similarly, SIAH1 protein expression was increased with a decrease in SLC25A6 in shXP2 HUVECs, which was accompanied by unchanged levels of mitophagy-associated proteins, PINK/Parkin and LC3B, indicating that the degradation of SLC25A6 was also independent of autophagy (Figure 6d). SIAH1 is an E3 ubiquitin protein ligase that mediates the ubiquitination and subsequent proteasomal degradation of its target proteins (Nagano et al., 2003; Liu et al., 2012). Therefore, we assumed that SIAH1 may be recruited to SLC25A6 for ubiquitination when XPNPEP2 is absent. A Co-IP assay confirmed that SLC25A6 and SIAH1 interact (Figure 6e), which was in agreement with their co-localization, as shown by immunofluorescence analysis with a Pearson’s correlation coefficient of 0.637 calculated from three independent replicates (n ≥ 30 cells per replicate) (Figure 6f). Furthermore, increased ubiquitination of SLC25A6 particularly occurred in shXP2 HUVECs and then was subsequently inhibited by MG132 treatment (Figure 6g). Undoubtedly, the degradation of SLC25A6 caused by XPNPEP2 ablation was rescued when MG132 was added (Figure 6h). Moreover, SIAH1 knockdown rescued the effect of shXP2 on SLC25A6 expression (Figure 6i), solidifying the interaction of SIAH1 and SLC25A6. Thus, our data here deciphered that XPNPEP2 ablation downregulated SLC25A6 via SIAH1-mediated proteasomal degradation.

### 3.6 Blocking SLC25A6 suppresses EC angiogenesis

**FIGURE 7:**
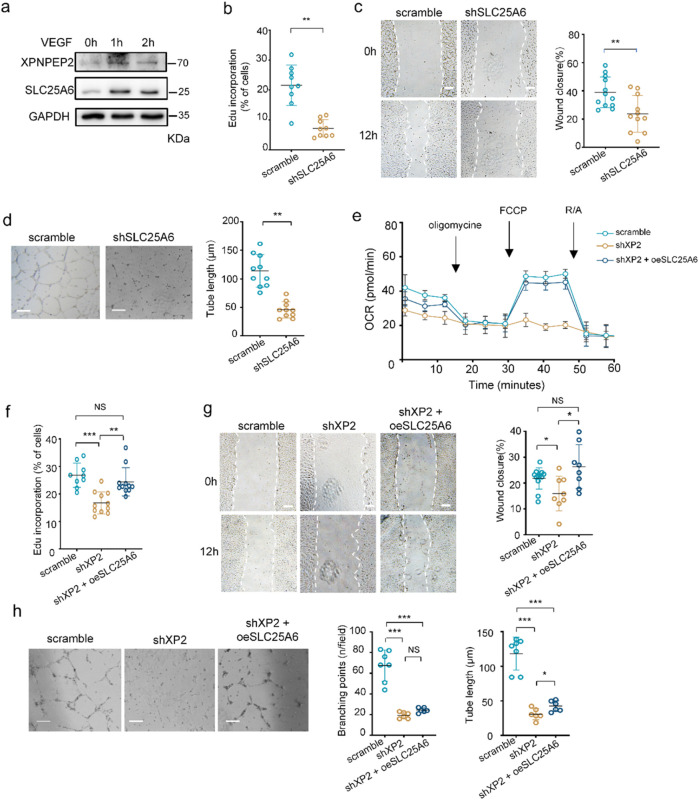
SLC25A6 deficiency suppresses EC angiogenesis. **(a)** Western blot analysis of XPNPEP2 and SLC25A6 in HUVECs with or without VEGF (50 ng/mL) treatment. GAPDH was used as the loading control. **(b)** Cell proliferation for shSLC25A6 and control HUVECs using the quantification of Edu incorporation. **(c)** Scratch wound assay for shSLC25A6 and control HUVECs after 12 h of culture and the quantification of wound closure; scale bar = 250 μm. **(d)** Tube formation for shSLC25A6 and control HUVECs and the quantification of tube length; scale bar = 500 μm. **(e)** Analysis of the rates of OCR in shXP2 HUVECs rescued by the overexpression of SLC25A6. Oligomycin, FCCP, and rotenone and antimycin A (R/A) were used as inhibitors. **(f)** Cell proliferation when SLC25A6 was overexpressed in shXPNPEP2 HUVECs. **(g)** Cell migration in shXPNPEP2 with SLC25A6 overexpression and the quantification of wound closure; scale bar = 250 μm. **(h)** Tube formation in shXPNPEP2 with SLC25A6 overexpression and the quantification branching points and tube length; scale bar = 500 μm. Data represent mean ± SD of triplicates. *, *P* < 0.05; **, *P* < 0.01; ***, *P* < 0.001; NS, no significance.

SLC25A6 is known for maintaining mitochondrial function. Since the ANGIOGENES data revealed SLC25A6 upregulation during early VEGF stimulation (Supplementary Figure S6a), we repeated the experiment in HUVECs and found similar results: XPNPEP2 and SLC25A6 were upregulated upon VEGF treatment for 1–2 h (Figure 7a). To investigate the angiogenic role of the XPNPEP2–SLC25A6 axis, we also efficiently downregulated SLC25A6 by shRNA in HUVECs and measured the three angiogenic parameters, namely, proliferation, migration, and tubulogenesis. As illustrated in Figures 7b–d, the three parameters were dramatically reduced by 14%, 15%, and 68%, respectively, relative to those of the controls, which was consistent with the data above from XPNPEP2 (Figures 3b–d). To confirm whether overexpressing SLC25A6 into shXP2 cells rescued mitochondrial morphology or/and respiratory chain function, we also carried out the TEM image and OCR assay in the oeSLC25A6 group. The vacuolated mitochondria were significantly decreased (Supplementary Figure S7a). Similarly, overexpressing SLC25A6 into shXP2 cells mostly rescued mitochondrial respiratory chain function (Figure 7e), whereas mROS was still increased into 129.7% compared with that of the controls (Supplementary Figure S7b). Moreover, the overexpression of SLC25A6 in shXP2 HUVECs notably restored EC proliferation and migration (Figures 7f,g) but limited improvement in tube formation (Figure 7h). Therefore, our data revealed that the loss of SLC25A6 impaired EC angiogenesis and that SLC25A6 overexpression could repair some of the capacity for angiogenesis suppressed by XPNPEP2 deficiency.

## 4 Discussion

The role of mitochondria in cellular processes extends far beyond energy production. Herein, we reveal a novel link between mitochondrial function and angiogenesis via modulation of the XPNPEP2–SLC25A6 axis. XPNPEP2 is essential for EC functions and regulates angiogenesis *in vivo* and *in vitro* via modulation of mitochondrial function. Silencing XPNPEP2 promoted the ubiquitination of SLC25A6 via SIAH1, resulting in mitochondrial dysfunction and therefore the inhibition of neovascularization.

Interestingly, XPNPEP2 is involved in mitochondria-mediated angiogenesis. XPNPEP2 deficiency inevitably leads to mitochondrial dysfunction, including reduced ATP, excessive mROS, and dramatic weakness of the respiratory chain, while it is partially distributed in the ER. The homologous protein of XPNPEP2, XPNPEP3, has a mitochondrial isoform and regulates the function of mitochondrial complex I (Tong et al., 2023). Neither XPNPEP3–XPNPEP2 nor XPNPEP2–MFN1 interaction was detected, and XPNPEP3 deficiency had no effect on EC proliferation (Supplementary Figure S8). Here, we found that SLC25A6, which usually facilitated ADP/ATP translocation and mPTP activity (Wu et al., 2020; Ji et al., 2023),was distributed partially in MAMs and interacted with XPNPEP2. Silencing XPNPEP2 promoted a notable reduction in the steady-state level of SLC25A6 by SIAH1-mediated proteasomal degradation, resulting in a reduction in MAMs, fragmentation of mitochondria, and collapse of mitochondrial function, which was concordant with recent observations that mitochondrial dysfunction and suppression of MAM formation led to inhibition of angiogenesis and tumor vascularization (Herkenne et al., 2020; Wang et al., 2021; Ma et al., 2023; Laplace and Bonneau, 2024). However, elongated mitochondria in response to angiogenic stimuli are associated with an early increase in Opa1 levels (Herkenne et al., 2020). The swelled, fragmented, and vacuolar mitochondria in shXP2 HUVECs were decreased by restoring XPNPEP2 overexpression, whereas overexpressing SLC25A6 into shXP2 HUVECs reduced vacuolar mitochondria and resulted in a slight improvement in mROS generation. A recent study reported that overexpression of SLC25A5 (ANT2), a homologous protein of SLC25A6, in aged mouse skin accelerated wound healing in the skin by increasing the ATP production rate (Woo et al., 2023). Here, as a downstream targeted protein in the XPNPEP2 network, overexpression of SLC25A6 failed to improve tube formation in XPNPEP2-ablated HUVECs, suggesting multiple functions of XPNPEP2, such as its enzymatic activity.

The mouse retinal vasculature expands in the postnatal period, which is a complex regulatory process. The superficial blood vessels extending radially from the optic papilla generally undergo a similar process of germination, growth, integration, stabilization, and pruning, including trunk vessel lengthening and peripheral microvascular remodeling, with the proximal region near the optic papilla being the first to complete this process (Selvam et al., 2018). Our present data exhibited that XPNPEP2 was essential for EC migration and branching. However, little change was observed in the radial expansion of the retinal vascular plexus in *Xpnpep2* ^*KO*^ pups on postnatal day 5, indicating the complex integration of multiple signals during angiogenesis. Nevertheless, the vascular defects in *Xpnpep2* ^KO^ retinas exhibited a distinct arterial–venous asymmetry, primarily decreased venous blood vessel density and branching in the proximal region of the superficial retinal vessels, suggesting that venous ECs were more susceptible to the mitochondrial dysfunction and cellular stress raised from XPNPEP2 deficiency. This observation aligns with the established biological disparity between arterial and venous ECs, which possess different developmental origins, gene expression profiles, and heterogeneity in the metabolic requirements during vascular remodeling (Li et al., 2019; Trimm and Red-Horse, 2023). Additionally, XPNPEP2 defects impeded the sprouting of distal tip cells. These vascular phenotypes in mice are consistent with hypertension-related microvascular structural remodeling and dysfunction (Humar et al., 2009), suggesting that the effects of XPNPEP2 on EC function and angiogenesis could explain some of the etiology of cardiovascular diseases caused by XPNPEP2 defects.

XPNPEP2 deletion prevented injury-induced neoangiogenesis without affecting quiescent vasculature under homeostatic conditions, which is in line with the recent notion that ECs undergo a complicated metabolic program, whereby different subtypes of ECs have different energetic demands during angiogenesis (Cruys et al., 2016; Luo et al., 2023). Although ECs are considered to perform glycolytic metabolism, our findings provide further evidence that mitochondrial OXPHOS is equally critical for the angiogenic response of ECs, as previously reported (Diebold et al., 2019; Li et al., 2019; Schiffmann et al., 2020). Glycolysis is postulated to meet the low energy requirements of vascular maintenance, while the energy consumption of ECs increases during wound healing and tumor growth (Li et al., 2019). Concordant results showed that the loss of XPNPEP2 leads to delayed wound healing and slow tumor growth due to abnormal vascularization, supporting that XPNPEP2 is involved in angiogenesis with high ATP demand.

The limitations of this study are as follows: although the inhibition of XPNPEP2 suppressed neoangiogenesis in LLC cells, a more comprehensive approach, such as spheroid angiogenesis, is essential for enhancing its potential as a therapeutic target for angiogenesis-related diseases. Conditional knockout mouse with endothelial-specific deletion of XPNPEP2, in which the Tie2-Cre or VE-cadherin-Cre driver was used, would provide more precise insights into its cell-autonomous role in vascular function. The mechanism through which SIAH1 is recruited to target proteins and its precise effects on angiogenesis need further investigation.

In summary, we identified a new role of XPNPEP2 in angiogenesis through the modulation of mitochondrial function. Inhibition of XPNPEP2 promotes SIAH1 expression to degrade SLC25A6, resulting in mitochondrial dysfunction and subsequent suppression of neoangiogenesis. Our findings highlight the importance of XPNPEP2-mediated mitochondrial function in angiogenesis, which may suggest a potential candidate target for the treatment of angiogenesis-related diseases.

## Data Availability

The datasets presented in this study can be found in online repositories. The names of the repository/repositories and accession number(s) can be found in the article/Supplementary Material.
