# Supplementary material for: XPNPEP2 regulates angiogenesis via modulation of mitochondrial function through SLC25A6
Source: Front Cell Dev Biol. 2026 Jan 7;13:1698651. doi: 10.3389/fcell.2025.1698651 (PMC12819664; doi:10.3389/fcell.2025.1698651)
Supplement: Supplementary file 1 [file DataSheet1.pdf]

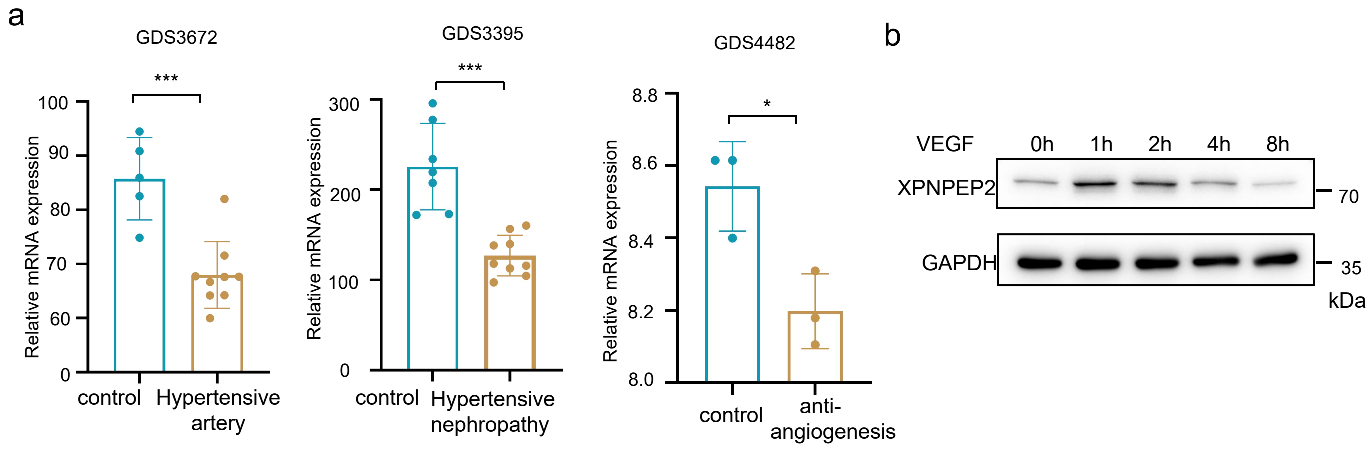

Supplementary figure 1 (a) Comparison of XPNPEP2 mRNA expression levels between diseased and controls mouse tissues in GDS3672, GDS3395, and GDS4482. (b) Protein levels of XPNPEP2 in responding to VEGF treatment in HUVECs. *, $P < 0.05$; **, $P < 0.01$; ***, $P < 0.001$.

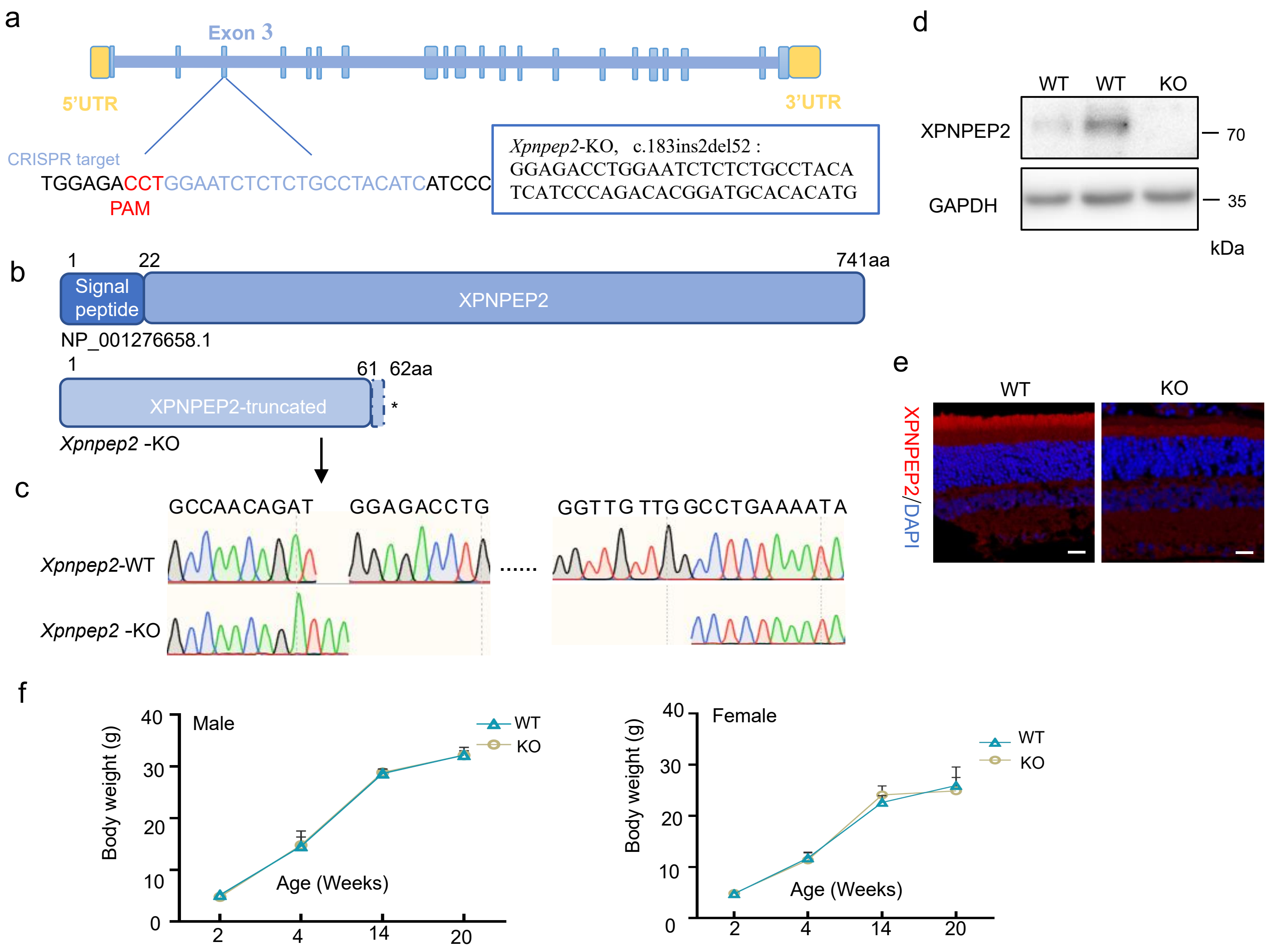

Supplementary figure 2 (a) Generation of Xpnpep2-knockout mouse using CRISPR/Cas9. Schematic of *Xpnpep2* gene. CRISPR target and PAM (Red) are located on Exon 3, selecting a clone with 2 bases insertion and 52 bases deletion at c.183(c.183ins2del52). (b) Schematic diagram of truncated Xpnpep2 protein. (c) Identification with Sanger sequencing. (d) Western blot analysis of Xpnpep2 in kidney tissues of *Xpnpep2*^{KO} and wild-type mice. GAPDH, used as a loading control. (e) Identification with immunofluorescence in retina of *Xpnpep2*^{KO} and wild-type mice. Bar: 30 μm. (f) Body weight between Xpnpep2^{WT} and Xpnpep2^{KO} mice under 20 weeks of age (n ≥ 6/genotype).*, $P < 0.05$, ***, $P < 0.001$.

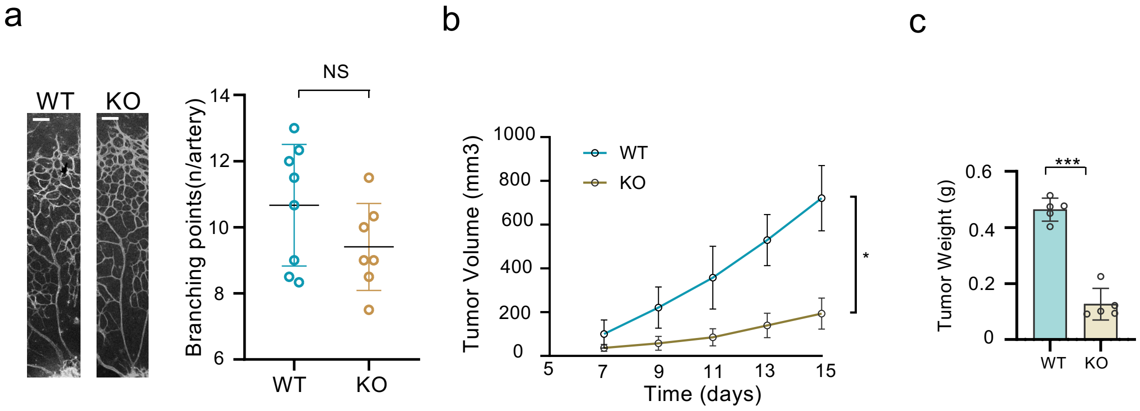

Supplementary figure 3 (a) Branching of retinal arteries, and the quantification of branching points. ns, not significant. Bar: 100 µm. (b) The volumes of LLC tumors in *Xpnpep2*^{KO} and *Xpnpep2*^{WT} mice. (c) The weights of LLC tumors in *Xpnpep2*^{KO} and *Xpnpep2*^{WT} mice. The data were recorded 15 days after injection. *, *P* < 0.05; **, *P* < 0.01; ***, *P* < 0.001, NS, not significant. n = 5/each genotype.

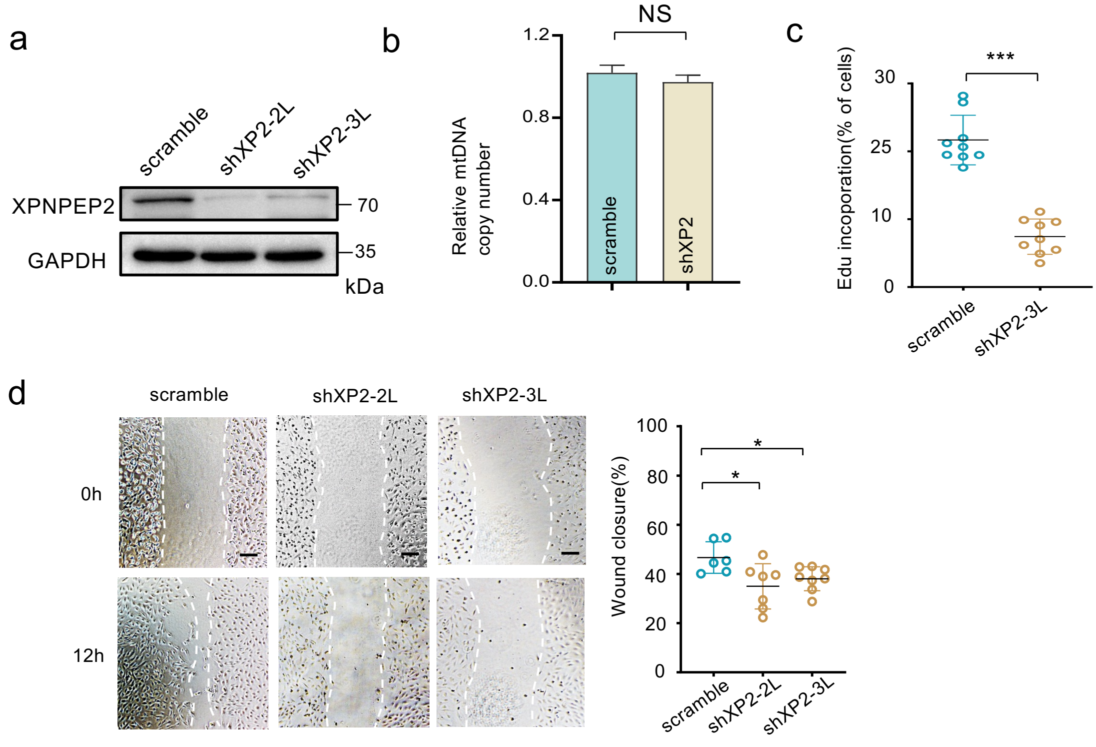

Supplementary figure 4 (a) Western blot analysis of XPNPEP2 in knockdown and control HUVECs. GAPDH, used as a loading control. (b) Analysis of mitochondrial DNA (mtDNA) copy number. genes used in qPCR: *mt-ND1*, normalized to the nuclear gene *B2M*. Data were present as the mean ± SD of triplicates. (c) (Cell proliferation in shXPNPEP2 and control HUVECs with Edu incorporation. (d)Scratch wound assay of shXPNPEP2 and control HUVECs after 12h culturing, and the quantification of wound closure. Bar,250 µm. *, *P* < 0.05; **, *P* < 0.01; ***, *P* < 0.001, NS, no significance.

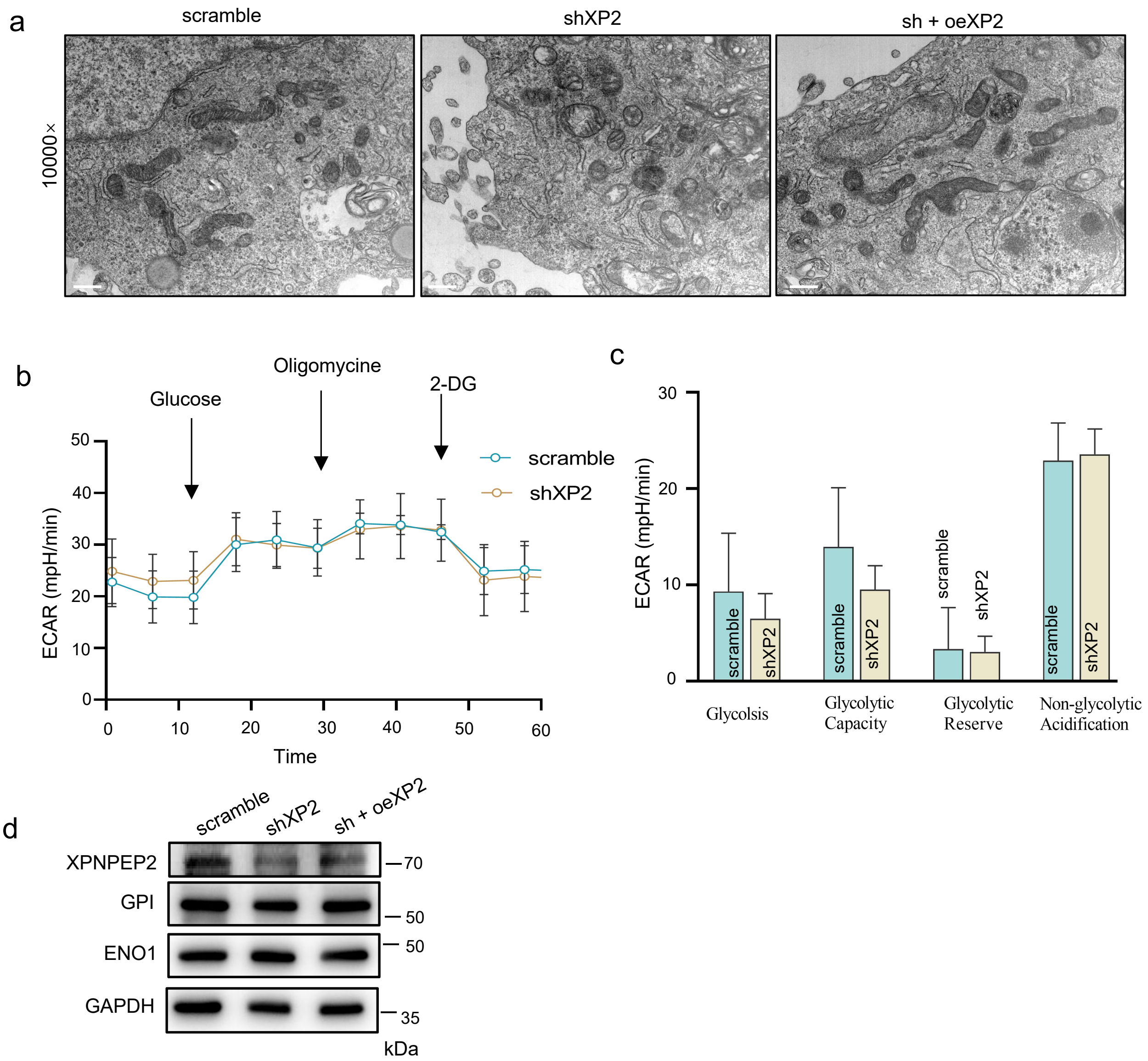

Supplementary figure 5 (a)) Mitochondria morphology by TEM in HUVECs. Bar: 0.5 μm. (b) Extracellular acidification rate (ECAR) in shXPNPEP2and control HUVECs using different inhibitors. Glycolysis was determined as increased ECAR after glucose. (c) Quantitative data of glycolysis, glycolytic capacity (determined as maximal ECAR after oligomycin), glycolytic reserve (determined as glycolytic capacity minus glycolysis.), and non-glycolytic acidification ECAR. (d) Western blot analysis of GPI and ENO1(relating to glycolysis) in shXPNPEP2 and control HUVECs, as well as in shXPNPEP2 HUVECs with overexpression of XPNPEP2 back. GAPDH, used as a loading control.

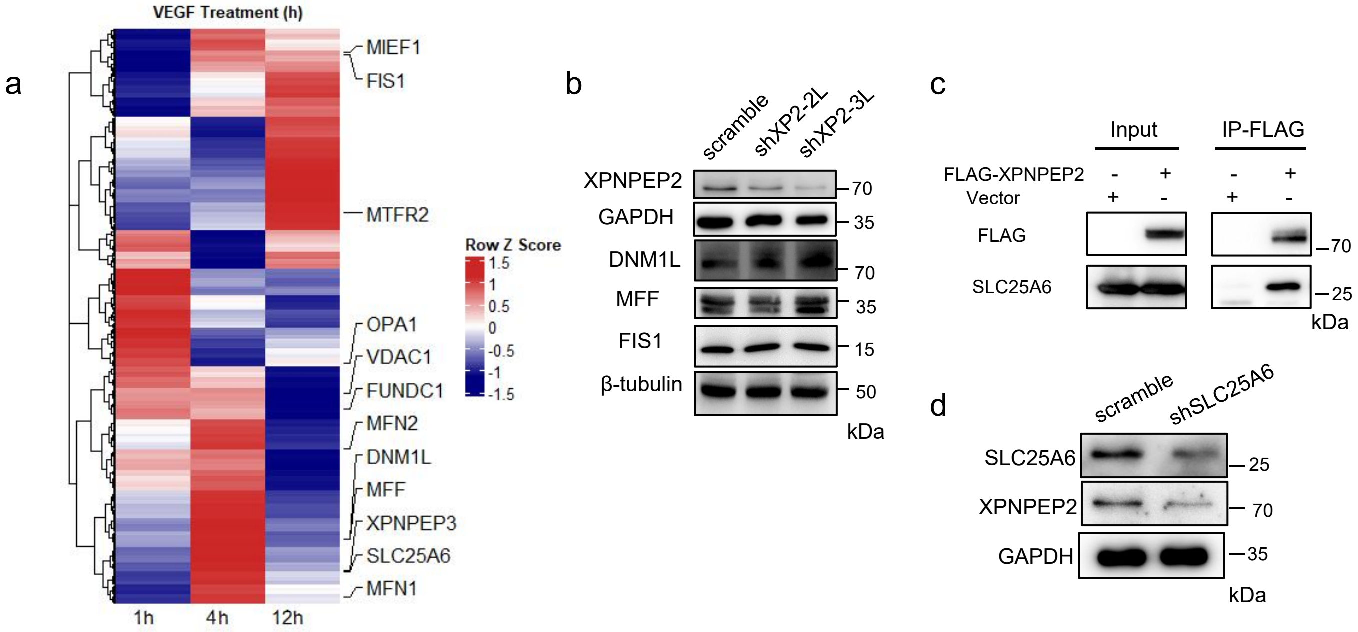

Supplementary figure 6 (a) Heatmap of transcriptional changes during VEGF treatment in HUVECs. Data are extracted from http:// angiogenes.uni-frankfurt.de and elaborated as detailed in the text. (b) Western blot analysis of proteins (MFF, DNM1L and FIS1) that associated with MAMs and mitochondrial dynamics in shXPNPEP2 and control HUVECs. β-actin or β-tubulin, used as a loading control. ShXP2-2L, -3L, for different clones of shXPNPEP2. (c) Co-immunoprecipitation of Flag-XPNPEP2 with SLC25A6 in HEK293T. (d)Reduction of XPNPEP2 in shSLC25A6 HUVECs.

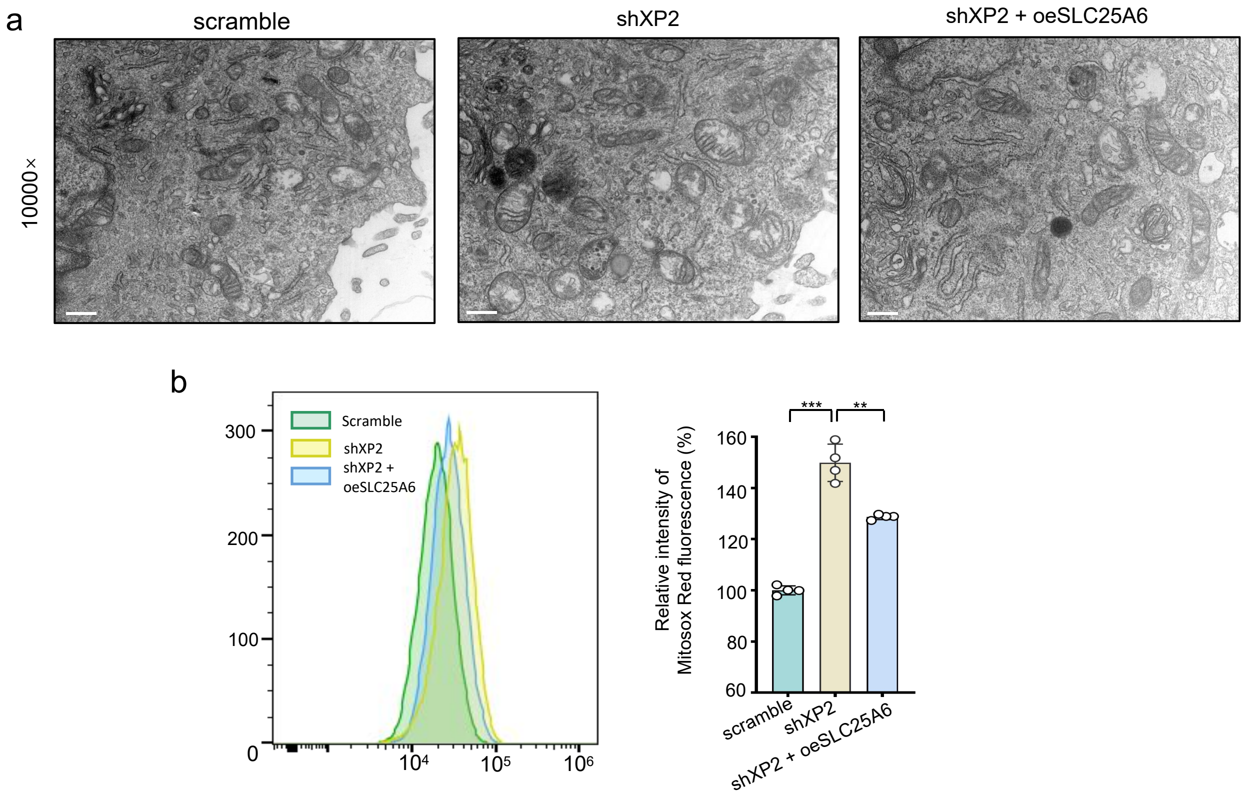

Supplementary figure 7 (a) Mitochondria morphology by TEM in HUVECs when XPNPEP2 is absent and SLC25A6 is overexpressed for rescue. Bar: 0.5 μm. (b) Mitochondrial ROS in HUVECs when XPNPEP2 is absent and SLC25A6 is overexpressed for rescue. ROS in shXP2, 149.8%; ROS in oeSLC25A6, 129.7%. **, *P* < 0.01; ***, *P* < 0.001.

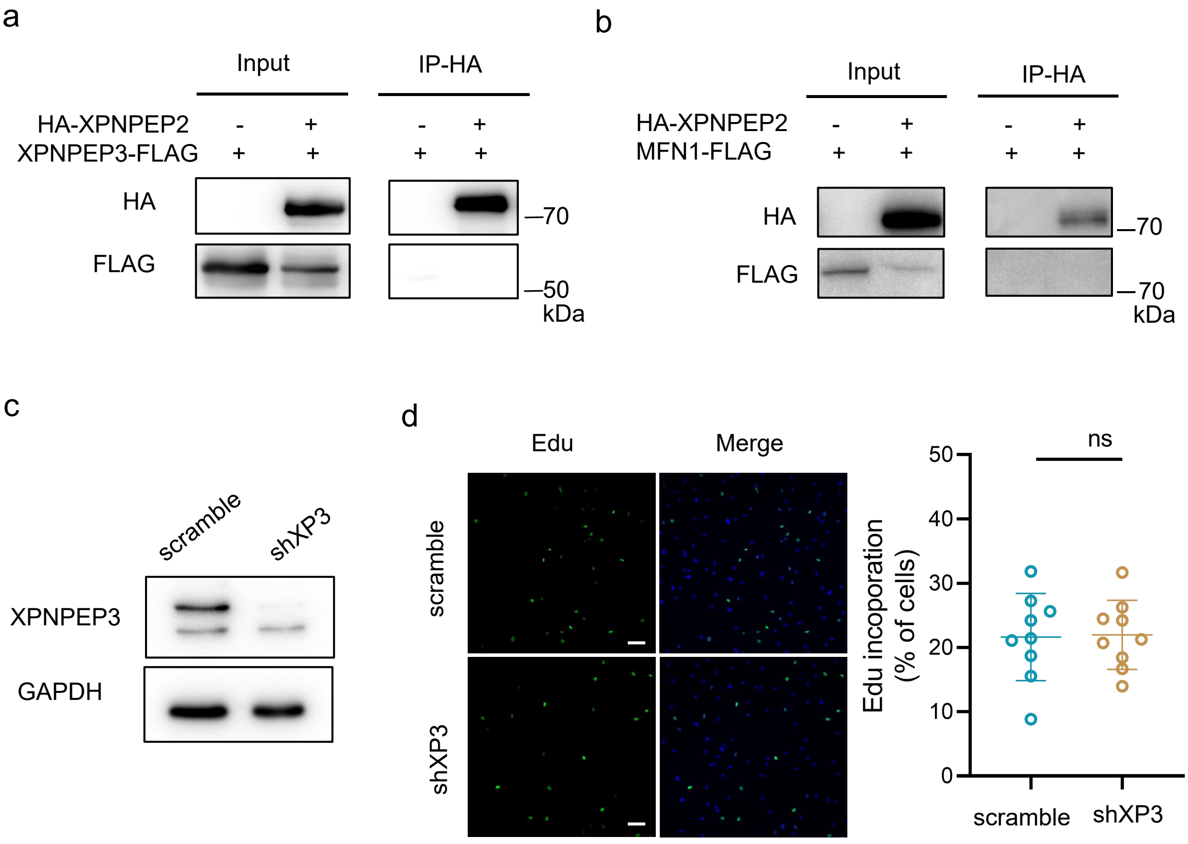

Supplementary figure 8. (a) Co-immunoprecipitation of HA-XPNPEP2 with XPNPEP3-FLAG or (b) MFN1-FLAG. (c) Western blot analysis of XPNPEP3 expression in shXPNPEP3 and control HUVECs. GAPDH, used as a loading control. (d) HUVECs proliferation using Edu incorporation in control and shXP3 groups. ns, no significance.

Supplemental table 1. shRNA primers and qPCR primers

**Primers**	**Oligo bases（5’---3’）**
Scramble-F	CCGGAAGCTTCGCGCGTAGTCTTACTCGAGTAAGACTACGGCGCGAAGCTTTTTTTG
Scramble-R	AATTCAAAAATAAGACTACGGCGCGAAGCCTCGAGAAGCTTCGCGCGTAGTCTTA
shXP2-2LF	CCGGCCAGTACCTGAATCGCTACTACTCGAGTAGTAGCGATTCAGGTACTGGTTTTTG
shXP2-2LR	AATTCAAAAACCAGTACCTGAATCGCTACTACTCGAGTAGTAGCGATTCAGGTACTGG
shXP2-3LF	CCGGAGCAGATGCAGACCCAGAATCCTCGAGGATTCTGGGTCTGCATCTGCTTTTTG
shXP2-3LR	AATTCAAAAAAGCAGATGCAGACCCAGAATCCTCGAGGATTCTGGGTCTGCATCTGCT
shXP2-4LF	CCGGCGAAACCTTGAGCTATCTGAACTCGAGTTCAGATAGCTCAAGGTTTCGTTTTTG
shXP2-4LR	AATTCAAAAACGAAACCTTGAGCTATCTGAACTCGAGTTCAGATAGCTCAAGGTTTCG
shSLC25A6-1LF	CCGGCATCGTGGTGAGCTGGATGATCTCGAGATCATCCAGCTCACCACGATGTTTTTG
shSLC25A6-1LR	AATTCAAAAACATCGTGGTGAGCTGGATGATCTCGAGATCATCCAGCTCACCACGATG
shSLC25A62LF	CCGGCGACAAGCAGTACAAGGGCATCTCGAGATGCCCTTGTACTGCTTGTCGTTTTTG
shSLC25A6-2LR	AATTCAAAAACGACAAGCAGTACAAGGGCATCTCGAGATGCCCTTGTACTGCTTGTCG
shMFN1-1LF	CCGGGCTCAAAGTTGTAAATGCTTTCTCGAGAAAGCATTTACAACTTTGAGCTTTTTG
shMFN1-1LR	AATTCAAAAAGCTCAAAGTTGTAAATGCTTTCTCGAGAAAGCATTTACAACTTTGAGC
shMFN1-2LF	CCGGGCTCCCATTATGATTCCAATACTCGAGTATTGGAATCATAATGGGAGC TTTTTG
shMFN1-2LR	AATTCAAAAAGCTCCCATTATGATTCCAATACTCGAGTATTGGAATCATAATGGGAGC
shXP3-1LF	CCGGCCTGGGATGGTAATCACAATTCTCGAGAATTGTGATTACCATCCCAGGTTTTTG
shXP3-1LR	AATTCAAAAACCTGGGATGGTAATCACAATTCTCGAGAATTGTGATTACCATCCCAGG
shXP3-2LF	CCGGAGGACTATCTCAGGTGGAATACTCGAGTATTCCACCTGAGATAGTCCTTTTTG
shXP3-2LR	AATTCAAAAAAGGACTATCTCAGGTGGAATACTCGAGTATTCCACCTGAGATAGTCCT
shSIAH1	GATCGGCTTACCGACTTGAGCTAAATCTCGAGATTTAGCTCAAGTCGGTAAGCTTTTTT
GAPDH-F	GGAGCGAGATCCCTCCAAAAT
GAPDH-R	GGCTGTTGTCATACTTCTCATGG

CTF1-F	AGGGAAGTCTGGAAGACCCC
CTF1-R	AGCTGCTCAGCGTATTTGGT
LIF-F	CCAACGTGACGGACTTCCC
LIF-R	TACACGACTATGCGGTACAGC
CYP2J2-F	TGGCTTGCCCTTAATCAAAGAA
CYP2J2-R	GGCCACTTGACATAATCAATCCA
PRKCQ-F	ATGTCGCCATTTCTTCGGATT
PRKCQ-R	ACATACTCTTTGACGAGCACAG
HSPA9-F	CTTGTTTCAAGGCGGGATTATGC
HSPA9-R	GCAGGAGTTGGTAGTACCCAAA
TIMM13-F	CAGAGGATGACGGACAAGTGT
TIMM13-R	CATGTAGCGGTCCATGCACA
XPNPEP2-F	GGTCAATACCACAATGTCACTCA
XPNPEP2-R	ACGCCTCTCGTCATGTTGG
B2M-F	GAGGCTATCCAGCGTACTCCA
B2M-R	CGGCAGGCATACTCATCTTTT
MTND1-F	CCACCTCTAGCCTAGCCGTTTA
MTND1-R	GGGTCATGATGGCAGGAGTAAT

Supplemental table 2. Antibodies used in the study

**Antibodies**	**Source and identifier**
XPNPEP2	Abcam（ab97852）
XPNPEP2	Abclonal（A10255）
SLC25A6(ANT3)	Proteintech（14841-1-AP）
MFN1	Proteintech（13798-1-AP）
MFN2	Abcam（ab186317）
DNM1L	Proteintech(12957-1-AP)
MFF	Proteintech(17090-1-AP)
FIS1	Abclonal（A19666）
TOM20	Proteintech (11802-1-AP)
BIP	Proteintech (11587-1-AP)
ACE	Proteintech（24743-1-AP）
GPI	Abclonal（A13308）
ENO1	Proteintech（11204-1-AP）
SIAH1	Proteintech (13886-1-AP)
	Thermo (PA5-88583)
PINK	Abcam（ab216144）
PARKIN	Proteintech（14060-1-AP）
LC3B	Abclonal（A19665）
Ub	CST（#3936）
GAPDH	Proteintech（60004-1-Ig）
Tubulin	Proteintech（6603KO1-Ig）
VDAC	Proteintech（10866-1-AP）
Anti HA-Tag	Abclonal（AE008）
Anti DDDDK-Tag	Abclonal（AE005）
Anti HA-Tag	Abcam（ab137838）
Goat Anti-Mouse IgG H&L (Alexa Fluor® 488)	Abcam（ab150113）
Goat Anti-Rabbit IgG H&L (Alexa Fluor® 594)	Abcam（ab150080）
CD31	BioLegend（WM59）

Supplemental table 3. mitochondrial genes changed during VEGF stimulation in ANGIOGENES

code	gene	code	gene	code	gene
V1	AARS2	V43	LIG3	V85	MRPL48
V2	ALKBH1	V44	LRPPRC	V86	MRPL50
V3	ANGEL2	V45	METAP1D	V87	MRPL51
V4	APEX1	V46	METTL15	V88	MRPL52
V5	ATAD3A	V47	METTL17	V89	MRPL53
V6	ATAD3B	V48	METTL4	V90	MRPL54
V7	AURKAIP1	V49	METTL5	V91	MRPL55
V8	C12orf65	V50	MGME1	V92	MRPL57
V9	CARS2	V51	MIEF1	V93	MRPL9
V10	CDK5RAP1	V52	MRPL1	V94	MRPS10
V11	CHCHD1	V53	MRPL10	V95	MRPS11
V12	COA3	V54	MRPL11	V96	MRPS12
V13	COX14	V55	MRPL13	V97	MRPS14
V14	DAP3	V56	MRPL14	V98	MRPS15
V15	DARS2	V57	MRPL15	V99	MRPS16
V16	DDX28	V58	MRPL16	V100	MRPS18A
V17	DHX30	V59	MRPL17	V101	MRPS18B
V18	DNA2	V60	MRPL18	V102	MRPS18C
V19	EARS2	V61	MRPL19	V103	MRPS2
V20	ELAC2	V62	MRPL2	V104	MRPS23
V21	ENDOG	V63	MRPL21	V105	MRPS24
V22	ERAL1	V64	MRPL22	V106	MRPS26
V23	EXD2	V65	MRPL23	V107	MRPS27
V24	EXOG	V66	MRPL24	V108	MRPS28
V25	FARS2	V67	MRPL27	V109	MRPS30
V26	FASTK	V68	MRPL28	V110	MRPS31
V27	FASTKD1	V69	MRPL3	V111	MRPS33
V28	FASTKD2	V70	MRPL30	V112	MRPS34
V29	FASTKD5	V71	MRPL32	V113	MRPS35
V30	GADD45GIP1	V72	MRPL34	V114	MRPS36
V31	GATB	V73	MRPL35	V115	MRPS5
V32	GFM1	V74	MRPL36	V116	MRPS7
V33	GRSF1	V75	MRPL37	V117	MRPS9
V34	GTPBP10	V76	MRPL38	V118	MRRF
V35	GTPBP3	V77	MRPL39	V119	MTERF1
V36	GUF1	V78	MRPL4	V120	MTERF3
V37	HARS2	V79	MRPL40	V121	MTERF4
V38	HEMK1	V80	MRPL41	V122	MTFMT
V39	HSD17B10	V81	MRPL42	V123	MTG1
V40	IARS2	V82	MRPL43	V124	MTG2
V41	LACTB2	V83	MRPL44	V125	MTIF2
V42	LARS2	V84	MRPL47	V126	MTIF3

V127	MTO1	V171	TIMM21	V215	IMMP2L
V128	MTPAP	V172	TOP1MT	V216	LACTB
V129	MTRF1	V173	TOP3A	V217	LAP3
V130	MTRF1L	V174	TRIT1	V218	MIPEP
V131	NARS2	V175	TRMT1	V219	MTX1
V132	NGRN	V176	TRMT10C	V220	MTX2
V133	NSUN2	V177	TRMT2B	V221	MUL1
V134	NSUN3	V178	TRMT5	V222	NLN
V135	NSUN4	V179	TRNT1	V223	OMA1
V136	OSGEPL1	V180	TRUB2	V224	PAM16
V137	OXA1L	V181	TSFM	V225	PARK7
V138	PARS2	V182	TUFM	V226	PARL
V139	PDE12	V183	UNG	V227	PHB
V140	PIF1	V184	VARS2	V228	PHB2
V141	PNPT1	V185	WARS2	V229	PITRM1
V142	POLB	V186	YARS2	V230	PMPCA
V143	POLDIP2	V187	YBEY	V231	PMPCB
V144	POLG	V188	YRDC	V232	PRSS35
V145	POLQ	V189	AFG3L2	V233	ROMO1
V146	POLRMT	V190	AGK	V234	SAMM50
V147	PPA2	V191	AIFM1	V235	SPG7
V148	PRIMPOL	V192	ATAD1	V236	STOML2
V149	PTCD3	V193	CASP3	V237	TIMM10
V150	PUS1	V194	CASP8	V238	TIMM10B
V151	QRSL1	V195	CASP9	V239	TIMM13
V152	QTRT1	V196	CHCHD4	V240	TIMM17A
V153	RARS2	V197	CLPB	V241	TIMM17B
V154	RBFA	V198	CLPP	V242	TIMM22
V155	RECQL4	V199	CLPX	V243	TIMM44
V156	REXO2	V200	DNAJA3	V244	TIMM50
V157	RMND1	V201	DNAJC11	V245	TIMM8A
V158	RNASEH1	V202	DNAJC15	V246	TIMM8B
V159	RPUSD3	V203	DNAJC19	V247	TIMM9
V160	SLIRP	V204	DNAJC4	V248	TOMM20
V161	SSBP1	V205	DNLZ	V249	TOMM22
V162	SUPV3L1	V206	FKBP10	V250	TOMM34
V163	TACO1	V207	GFER	V251	TOMM40
V164	TARS2	V208	GRPEL1	V252	TOMM40L
V165	TBRG4	V209	GRPEL2	V253	TOMM5
V166	TEFM	V210	HSPD1	V254	TOMM6
V167	TFAM	V211	HSPE1	V255	TOMM7
V168	TFB1M	V212	HTRA2	V256	TRAP1
V169	TFB2M	V213	IDE	V257	UQCRC1
V170	THG1L	V214	IMMP1L	V258	UQCRC2

V259	USP30	V281	COX5A	V303	NDUFA11
V260	XPNPEP3	V282	COX5B	V304	NDUFA12
V261	YME1L1	V283	COX6A1	V305	NDUFA13
V262	ACAD9	V284	COX6B1	V306	NDUFA2
V263	ATPAF1	V285	COX6B2	V307	NDUFA3
V264	ATPAF2	V286	COX6C	V308	NDUFA4
V265	BCS1L	V287	COX7A1	V309	NDUFA5
V266	CEP89	V288	COX7A2	V310	NDUFA6
V267	CMC1	V289	COX7A2L	V311	NDUFA8
V268	CMC2	V290	COX7B	V312	NDUFA9
V269	COA1	V291	COX7C	V313	NDUFAB1
V270	COA4	V292	COX8A	V314	NDUFAF1
V271	COA5	V293	CYC1	V315	NDUFAF2
V272	COA6	V294	CYCS	V316	NDUFAF3
V273	COA7	V295	ECSIT	V317	NDUFAF4
V274	COX10	V296	HCCS	V318	NDUFAF5
V275	COX11	V297	HIGD1A	V319	NDUFAF6
V276	COX15	V298	HIGD2A	V320	NDUFB1
V277	COX16	V299	LYRM2	V321	NDUFB10
V278	COX17	V300	LYRM7	V322	NDUFB11
V279	COX18	V301	NDUFA1	V323	NDUFB2
V280	COX4I1	V302	NDUFA10	V324	NDUFB3
V325	NDUFB4	V347	SCO2	V369	UQCRH
V326	NDUFB5	V348	SDHA	V370	UQCRQ
V327	NDUFB6	V349	SDHAF1	V371	AADAT
V328	NDUFB7	V350	SDHAF2	V372	AASS
V329	NDUFB8	V351	SDHB	V373	ABCB10
V330	NDUFB9	V352	SDHC	V374	ABCB7
V331	NDUFC1	V353	SDHD	V375	ABHD10
V332	NDUFC2	V354	SURF1	V376	ABHD11
V333	NDUFS1	V355	TIMMDC1	V377	ACAA1
V334	NDUFS2	V356	TMEM126A	V378	ACAA2
V335	NDUFS4	V357	TMEM126B	V379	ACACB
V336	NDUFS5	V358	TMEM177	V380	ACAD8
V337	NDUFS6	V359	TMEM186	V381	ACADM
V338	NDUFS7	V360	TMEM70	V382	ACADS
V339	NDUFS8	V361	TTC19	V383	ACADSB
V340	NDUFV1	V362	UQCC1	V384	ACADVL
V341	NDUFV2	V363	UQCC2	V385	ACAT1
V342	NDUFV3	V364	UQCC3	V386	ACLY
V343	NUBPL	V365	UQCR10	V387	ACO2
V344	PET100	V366	UQCR11	V388	ACOT11
V345	PNKD	V367	UQCRB	V389	ACOT2
V346	SCO1	V368	UQCRFS1	V390	ACOT7

V391	ACOT9	V435	CHCHD7	V479	ECI1
V392	ACP6	V436	CHDH	V480	ECI2
V393	ACSF2	V437	CHPT1	V481	EHHADH
V394	ACSF3	V438	CISD1	V482	EPHX2
V395	ACSL1	V439	CKMT1A	V483	ETFA
V396	ACSL6	V440	CLYBL	V484	ETFB
V397	ACSM4	V441	CMPK2	V485	ETFDH
V398	ACSM5	V442	COASY	V486	ETHE1
V399	ACSS1	V443	COMT	V487	FAHD1
V400	ACSS3	V444	COQ10A	V488	FAM210B
V401	AGPAT4	V445	COQ10B	V489	FASN
V402	AGPAT5	V446	COQ2	V490	FDPS
V403	AGXT	V447	COQ3	V491	FDX1
V404	AIFM2	V448	COQ4	V492	FDXR
V405	AK2	V449	COQ7	V493	FECH
V406	AK3	V450	COQ9	V494	FH
V407	AK4	V451	CPOX	V495	FHIT
V408	AKR1B10	V452	CPS1	V496	FLAD1
V409	AKR7A2	V453	CPT1A	V497	FPGS
V410	ALAS1	V454	CPT1C	V498	FTH1
V411	ALDH18A1	V455	CPT2	V499	FXN
V412	ALDH1B1	V456	CRLS1	V500	GATM
V413	ALDH1L1	V457	CROT	V501	GCAT
V414	ALDH1L2	V458	CS	V502	GCDH
V415	ALDH2	V459	CYB5B	V503	GCSH
V416	ALDH3A2	V460	CYB5R3	V504	GLDC
V417	ALDH4A1	V461	CYP27A1	V505	GLRX2
V418	ALDH6A1	V462	D2HGDH	V506	GLRX5
V419	ALDH7A1	V463	DBI	V507	GLS
V420	ALDH9A1	V464	DBT	V508	GLUD1
V421	AMT	V465	DCXR	V509	GLUD2
V422	AUH	V466	DECR1	V510	GLYAT
V423	BCAT2	V467	DGUOK	V511	GOT2
V424	BCKDHB	V468	DHODH	V512	GPAM
V425	BCKDK	V469	DHRS2	V513	GPAT2
V426	BDH1	V470	DHRS4	V514	GPD2
V427	BOLA1	V471	DHTKD1	V515	GPT2
V428	BOLA3	V472	DLAT	V516	GPX4
V429	BPHL	V473	DLST	V517	GRHPR
V430	CA5A	V474	DMGDH	V518	GSR
V431	CA5B	V475	DTYMK	V519	GSTK1
V432	CAT	V476	DUT	V520	GSTZ1
V433	CBR3	V477	ECHDC1	V521	GUK1
V434	CBR4	V478	ECHS1	V522	HADH

V523	HADHA	V567	MOCS1	V611	PCK2
V524	HADHB	V568	MPC1	V612	PDHA1
V525	HAGH	V569	MPC2	V613	PDHB
V526	HIBADH	V570	MPST	V614	PDHX
V527	HIBCH	V571	MSRA	V615	PDK2
V528	HINT1	V572	MSRB2	V616	PDK3
V529	HINT2	V573	MSRB3	V617	PDK4
V530	HMGCL	V574	MTHFD1L	V618	PDP1
V531	HSCB	V575	MTHFD2	V619	PDP2
V532	HSD17B4	V576	MTHFD2L	V620	PDSS2
V533	HSD17B8	V577	MTHFS	V621	PGS1
V534	HSPA9	V578	NADK2	V622	PHYH
V535	IBA57	V579	NAGS	V623	PNPLA8
V536	IDH2	V580	NAT8L	V624	PNPO
V537	IDH3A	V581	NEU4	V625	PPM1K
V538	IDH3B	V582	NFU1	V626	PPOX
V539	IDH3G	V583	NIT2	V627	PRDX2
V540	IDI1	V584	NME3	V628	PRDX3
V541	ISCA1	V585	NME4	V629	PRDX4
V542	ISCU	V586	NME6	V630	PRDX5
V543	IVD	V587	NMNAT3	V631	PRDX6
V544	L2HGDH	V588	NNT	V632	PRELID1
V545	LDHAL6B	V589	NT5DC2	V633	PRODH
V546	LDHB	V590	NT5M	V634	PTGES2
V547	LDHD	V591	NUDT13	V635	PYCR1
V548	LIAS	V592	NUDT19	V636	PYCR2
V549	LYPLA1	V593	NUDT2	V637	QDPR
V550	LYPLAL1	V594	NUDT5	V638	RDH13
V551	LYRM4	V595	NUDT8	V639	RFK
V552	MAOA	V596	NUDT9	V640	RPIA
V553	MCAT	V597	OAT	V641	RSAD1
V554	MCCC1	V598	OGDH	V642	SARDH
V555	MCCC2	V599	OGDHL	V643	SCP2
V556	MCEE	V600	OSBPL1A	V644	SDSL
V557	MDH2	V601	OXCT1	V645	SERAC1
V558	ME2	V602	OXCT2	V646	SFXN1
V559	ME3	V603	OXR1	V647	SFXN3
V560	MECR	V604	OXSM	V648	SFXN5
V561	MGST1	V605	PAICS	V649	SHMT2
V562	MGST3	V606	PANK2	V650	SIRT4
V563	MLYCD	V607	PC	V651	SIRT5
V564	MMAA	V608	PCBD2	V652	SLC25A1
V565	MMAB	V609	PCCA	V653	SLC25A10
V566	MMADHC	V610	PCCB	V654	SLC25A11

V655	SLC25A12	V699	ABCA9	V743	NLRX1
V656	SLC25A13	V700	ABCB8	V744	OCIAD2
V657	SLC25A14	V701	ABCD1	V745	PDE2A
V658	SLC25A15	V702	ABCD2	V746	PPIF
V659	SLC25A19	V703	ABCD3	V747	PPTC7
V660	SLC25A20	V704	LETMD1	V748	PRKACA
V661	SLC25A21	V705	MCU	V749	RHOT1
V662	SLC25A23	V706	MICU1	V750	RHOT2
V663	SLC25A24	V707	MICU2	V751	AHCYL1
V664	SLC25A25	V708	MICU3	V752	APOO
V665	SLC25A28	V709	MPV17	V753	APOOL
V666	SLC25A3	V710	MRS2	V754	ARL2
V667	SLC25A32	V711	MTCH1	V755	ARMC10
V668	SLC25A33	V712	MTCH2	V756	ARMCX1
V669	SLC25A37	V713	SFXN2	V757	ARMCX3
V670	SLC25A38	V714	SFXN4	V758	BAD
V671	SLC25A4	V715	SLC25A16	V759	BAK1
V672	SLC25A41	V716	SLC25A18	V760	BAX
V673	SLC25A42	V717	SLC25A27	V761	BBC3
V674	SLC25A44	V718	SLC25A35	V762	BCL2
V675	SLC25A5	V719	SLC25A39	V763	BCL2A1
V676	SLC25A52	V720	SLC25A40	V764	BCL2L1
V677	SLC25A6	V721	SLC25A43	V765	BCL2L10
V678	SOD1	V722	SLC25A45	V766	BCL2L11
V679	SOD2	V723	SLC25A46	V767	BCL2L13
V680	SPR	V724	SLC30A9	V768	BCL2L2
V681	SPTLC2	V725	SLC8B1	V769	BID
V682	STARD7	V726	SMDT1	V770	BIK
V683	SUCLA2	V727	UCP2	V771	BNIP3
V684	SUCLG1	V728	UCP3	V772	BNIP3L
V685	SUCLG2	V729	VDAC1	V773	BOK
V686	SUGCT	V730	VDAC2	V774	CHCHD2
V687	SUOX	V731	VDAC3	V775	CHCHD3
V688	TAMM41	V732	ADCY10	V776	CHCHD6
V689	THEM4	V733	AKAP1	V777	DIABLO
V690	TK2	V734	AKAP10	V778	DNM1L
V691	TMLHE	V735	BLOC1S1	V779	FIS1
V692	TRIAP1	V736	C1QBP	V780	FKBP8
V693	TSPO	V737	EFHD1	V781	FUNDC1
V694	TST	V738	FUNDC2	V782	GHITM
V695	TXN2	V739	IFI27	V783	IMMT
V696	TXNRD1	V740	LETM1	V784	MCL1
V697	TXNRD2	V741	MACROD1	V785	MFF
V698	ZADH2	V742	MAVS	V786	MFN1

V787	MFN2	V793	NBR1	V799	SPIRE1
V788	MGARP	V794	NIPSNAP1	V800	STX17
V789	MIEF2	V795	OPA1	V801	STYXL1
V790	MTFR1	V796	PMAIP1	V802	SYNJ2BP
V791	MTFR2	V797	RMDN3	V803	TMEM11
V792	MTX3	V798	SNAP29	V804	VPS13D

Supplemental table 4. Protein profile of immunoprecipitation with XPNPEP2

protein	Score	emPAI	protein	Score	emPAI
XPNPEP2	894	1.53	COPG2	72	0.03
HNRNPA2B1	513	3.58	CANX	71	0.09
HNRNPH1	386	0.98	HBB	70	0.77
RTCB	386	1.23	CSE1L	70	0.04
TUBA1B	347	0.87	DNAJA1	69	0.15
FUS	262	0.81	LMNB2	69	0.1
AGO2	251	0.48	SRSF7	68	0.12
TUBB	250	0.89	RPS13	67	0.44
DDX3X	250	0.42	RBM14	67	0.05
HSPA5	239	0.95	RPL7	66	0.14
DHX9	225	0.31	TNRC6A	66	0.03
HNRNPU	194	0.33	FGG	64	0.13
HNRNPUL1	186	0.35	HNRNPH3	63	0.09
DDX1	183	0.42	PHGDH	61	0.27
TMEM263	167	2.6	HSPD1	61	0.05
DAZAP1	147	0.34	SNRPD2	60	0.95
EWSR1	144	0.28	HRNR	60	0.02
CLTC	139	0.12	CAND1	60	0.07
SLC25A6	134	0.95	RPL23	60	0.23
HNRNPD	133	0.7	PABPC1	60	0.1
TNRC6B	129	0.14	CAD	60	0.1
HNRNPK	125	0.28	HSPE1-MOB4	59	0.11
HNRNPC	125	0.34	PCBP2	59	0.21
NONO	122	0.42	RPL4	59	0.07
TFG	120	0.55	SFXN2	58	0.09
C14orf166	109	0.4	NPM1	57	0.1
LMNB1	106	0.24	HIST1H1D	56	0.32
FAM98A	105	0.12	HBA2	56	0.29
RPL8	104	0.32	RPL35	56	0.23
PDIA6	103	0.14	MTHFD1	56	0.03
SLC25A11	102	0.34	SFN	55	0.12
SERBP1	98	0.15	ERH	55	0.28
EEF1A1P5	97	0.29	YBX2	55	0.09
TIA1	93	0.25	SNRPG	54	0.41
VIM	89	0.29	SFPQ	54	0.09

HSPA9	85	0.09	RPS4X	53	0.11
RBMX	84	0.49	RPL13	52	0.14
ENO1	82	0.22	RPS15A	51	0.23
RPL18	82	0.53	TXN	51	0.29
Tim13	79	0.32	RPL3	51	0.23
RPL21	74	0.18	SMARCC1	51	0.24
KBTBD3	51	0.05	SRSF3	34	0.24
RPS23	50	0.78	SH3GLB2	34	0.07
RPS28	50	0.45	TRIM21	34	0.12
MIF	50	0.27	MATR3	33	0.31
IGLL5	50	0.14	ILF2	33	0.08
FGB	49	0.11	ABCA5	33	0.02
H1FX	49	0.15	RPL18A	33	0.16
HSP90AB1	49	0.08	IGHM	32	0.07
RPSAP58	49	0.1	PLCH1	32	0.02
SNRNP70	48	0.06	TCP1	32	0.09
SNRPA	47	0.17	MCM7	32	0.04
TMPO	46	0.12	HNRNPL	32	0.06
RPL37A	46	0.45	SNRPN	31	0.13
RPS19	46	0.21	SIPA1L1	31	0.02
RPN1	46	0.1	RPL23A	30	0.19
HIST1H2AB	45	0.54	CNN3	30	0.17
DHX29	46	0.02	SLC25A3	30	0.09
HNRNPM	37	0.09	RPS3A	28	0.14
ILF3	44	0.07	KHDRBS1	28	0.07
SS18	44	0.56	UBA52	28	0.5
DPF2	44	0.07	CTPS1	28	0.09
RPL30	44	0.27	CNN2	26	0.14
RPL10	42	0.14	CAPRIN1	26	0.05
RPL29	41	0.42	HIST1H4A	26	0.3
HNRNPR	41	0.09	SYNE1	25	
RPS2	41	0.15	PCNXL4	24	0.1
RPS5	39	0.23	RPL9	24	0.16
RPL34	39	0.25	ALB	23	0.05
SMARCA2	38	0.02	HSP90B1	23	0.04
RPS16	38	0.23	SMARCD2	22	0.06
TPI1	38	0.11	IMPDH2	22	0.06
RPS6	37	0.33	DUSP3	22	0.24
ARID1A	37	0.03	TIAM2	22	0.03
RPS9	31	0.32	PROSC	21	0.2
PRKDC	37	0.01	PDIA3	19	0.06
RPS3	37	0.13	ARHGEF18	19	0.03
CCT8	37	0.3	RPL32	18	0.22
SLC25A1	36	0.1	RPL6	18	0.21
TRIP11	36	0.01	ATP5A1	17	0.05

RPLP0	35	0.18	DDR1	16	0.18
ELAVL1	35	0.08			

Supplemental table 5. Genes differentially expressed from RNA-seq data analysis between control and shXP2 HUVECs

Name	log2FoldChange	pval	Regulation	Description
PRKCQ	-1.725330486	0.003000746	Down Regulation	protein kinase C theta [Source:HGNC Symbol;Acc:HGNC:9410]
H2AC20	-1.626937574	0.008395699	Down Regulation	H2A clustered histone 20 [Source:HGNC Symbol;Acc:HGNC:4738]
LIF	-1.718505229	0.01475548	Down Regulation	LIF interleukin 6 family cytokine [Source:HGNC Symbol;Acc:HGNC:6596]
EID2B	-1.591981807	0.031736281	Down Regulation	EP300 interacting inhibitor of differentiation 2B [Source:HGNC Symbol;Acc:HGNC:26796]
PDCD2L	-1.077358275	0.032155894	Down Regulation	programmed cell death 2 like [Source:HGNC Symbol;Acc:HGNC:28194]
FMO4	-2.439785545	0.036411085	Down Regulation	flavin containing dimethylaniline monoxygenase 4 [Source:HGNC Symbol;Acc:HGNC:3772]
FCMR	-3.489664199	0.047698103	Down Regulation	Fc mu receptor [Source:HGNC Symbol;Acc:HGNC:14315]
SEPTIN4	3.09798586	0.004195738	Up Regulation	septin 4 [Source:HGNC Symbol;Acc:HGNC:9165]
BORCS7	1.025307504	0.006329446	Up Regulation	BLOC-1 related complex subunit 7 [Source:HGNC Symbol;Acc:HGNC:23516]
MANEAL	2.466558843	0.016836581	Up Regulation	mannosidase endo-alpha like [Source:HGNC Symbol;Acc:HGNC:26452]
TTLL3	1.054321811	0.023055148	Up Regulation	tubulin tyrosine ligase like 3 [Source:HGNC Symbol;Acc:HGNC:24483]
TMEM30B	1.486999559	0.024919218	Up Regulation	transmembrane protein 30B [Source:HGNC Symbol;Acc:HGNC:27254]
GPR160	1.049772417	0.026683132	Up Regulation	G protein-coupled receptor 160 [Source:HGNC Symbol;Acc:HGNC:23693]
TSPAN10	1.512010529	0.02699425	Up Regulation	tetraspanin 10 [Source:HGNC Symbol;Acc:HGNC:29942]
C2orf88	2.16732527	0.029421216	Up Regulation	chromosome 2 open reading frame 88 [Source:HGNC Symbol;Acc:HGNC:28191]
SULF1	1.229190562	0.034268722	Up Regulation	sulfatase 1 [Source:HGNC Symbol;Acc:HGNC:20391]
SIAH1	1.050806394	0.035492056	Up Regulation	siah E3 ubiquitin protein ligase 1 [Source:HGNC Symbol;Acc:HGNC:10857]
CTF1	1.13563479	0.040088763	Up Regulation	cardiotrophin 1 [Source:HGNC Symbol;Acc:HGNC:2499]
TTBK1	2.024157499	0.040252964	Up Regulation	tau tubulin kinase 1 [Source:HGNC Symbol;Acc:HGNC:19140]
MRPS5	1.222537311	0.040738316	Up Regulation	mitochondrial ribosomal protein S5 [Source:HGNC Symbol;Acc:HGNC:14498]
ZNF571	2.338615135	0.041432572	Up Regulation	zinc finger protein 571 [Source:HGNC Symbol;Acc:HGNC:25000]
HOXC10	2.034922045	0.044877976	Up Regulation	homeobox C10 [Source:HGNC Symbol;Acc:HGNC:5122]
CLSTN2	3.683016656	0.046177141	Up Regulation	calsyntenin 2 [Source:HGNC Symbol;Acc:HGNC:17448]
